# Downregulation of the Host Gene *jigr1* by miR-92 Is Essential for Neuroblast Self-Renewal in *Drosophila*

**DOI:** 10.1371/journal.pgen.1005264

**Published:** 2015-05-22

**Authors:** Yeliz Yuva-Aydemir, Xia-Lian Xu, Ozkan Aydemir, Eduardo Gascon, Serkan Sayin, Wenke Zhou, Yang Hong, Fen-Biao Gao

**Affiliations:** 1 Department of Neurology, University of Massachusetts Medical School, Worcester, Massachusetts, United States of America; 2 Gladstone Institute of Neurological Disease, San Francisco, California, United States of America; 3 Department of Cell Biology, University of Pittsburgh, Pittsburgh, Pennsylvania, United States of America; Stanford University School of Medicine, UNITED STATES

## Abstract

Intragenic microRNAs (miRNAs), located mostly in the introns of protein-coding genes, are often co-expressed with their host mRNAs. However, their functional interaction in development is largely unknown. Here we show that in *Drosophila*, *miR-92a* and *miR-92b* are embedded in the intron and 3’UTR of *jigr1*, respectively, and co-expressed with some *jigr1* isoforms. miR-92a and miR-92b are highly expressed in neuroblasts of larval brain where Jigr1 expression is low. Genetic deletion of both *miR-92a* and *miR-92b* demonstrates an essential cell-autonomous role for these miRNAs in maintaining neuroblast self-renewal through inhibiting premature differentiation. We also show that miR-92a and miR-92b directly target *jigr1* in vivo and that some phenotypes due to the absence of these miRNAs are partially rescued by reducing the level of *jigr1*. These results reveal a novel function of the miR-92 family in *Drosophila* neuroblasts and provide another example that local negative feedback regulation of host genes by intragenic miRNAs is essential for animal development.

## Author Summary

Animal development is regulated by many genes including a class of small RNAs called microRNAs (miRNAs). Nearly half of the miRNAs are located in the protein coding genes but functional importance of this genomic organization is unclear. Here we use *Drosophila* stem cells in the brain as a model system to investigate the interactions between miR-92a and miR-92b and their host gene *jing interacting regulatory protein 1 (jigr1)*. Our studies reveal that these miRNAs prevent premature differentiation of neural stem cells and they do so in part through directly targeting and suppressing their host gene, *jigr1*. Our results reveal a novel function of the miR-92 family and identify another negative feedback loop as an essential regulator in neural stem cell development.

## Introduction

MicroRNAs (miRNAs) are short (~21–23 nt) noncoding RNAs that regulate gene expression post-transcriptionally in many physiological and pathological processes [1,2]. In the canonical miRNA biogenesis pathway, a long primary transcript (pri-miRNA) is generated by RNA polymerase II and cleaved by a nuclear complex formed by Drosha and DGCR8 [3]. Some pri-miRNAs produce miRNAs only (intergenic miRNAs) while others contain miRNAs in the intronic regions of protein-coding “host” genes (intragenic miRNAs) [4]. Many intronic miRNAs and host gene mRNAs are likely co-expressed [5,6] but others may not be [7,8]. Few cases have been experimentally confirmed, and the functional significance of such a genomic arrangement is largely unknown.

In this study, we used the differentiation of *Drosophila* neuroblasts as a model system to examine the expression and function of specific miRNAs. *Drosophila* neuroblasts form during embryonic development and enter a proliferative quiescent state at the end of embryogenesis [9]. In the early larval stage, neuroblasts reenter the cell cycle and undergo a series of proliferative symmetric and self-renewing asymmetric cell divisions to maintain the progenitor pool and generate diverse cell types [10,11]. In each asymmetric cell division, neuroblasts divide to generate a neuroblast cell and a ganglion mother cell, which divides only once to generate two neurons or one neuron and one glial cell. The balance between self-renewal and differentiation is critical for normal development, but the mechanisms are incompletely understood [12].

Here we show that the gene encoding jing-interacting gene regulatory 1 (*jigr1*), a putative DNA-binding protein containing MADF domain with unknown function [13], hosts *miR-92a* in the intron and *miR-92b* in the 3’UTR. We also uncover the functional significance of this intragenic miRNA–host gene interaction through genetic knockout of both miR-92a and miR-92b. During larval development, miR-92 family limits *jigr1* expression in neuroblasts and is essential for maintenance of a neuroblast pool. We propose that this genomic arrangement and local feed-back regulatory loop are essential for animal development to ensure the generation of the proper number of neuronal and glial cells.

## Results

### miR-92a and miR-92b Are Expressed in Neuroblasts of the *Drosophila* Larval Brain

**Fig 1:**
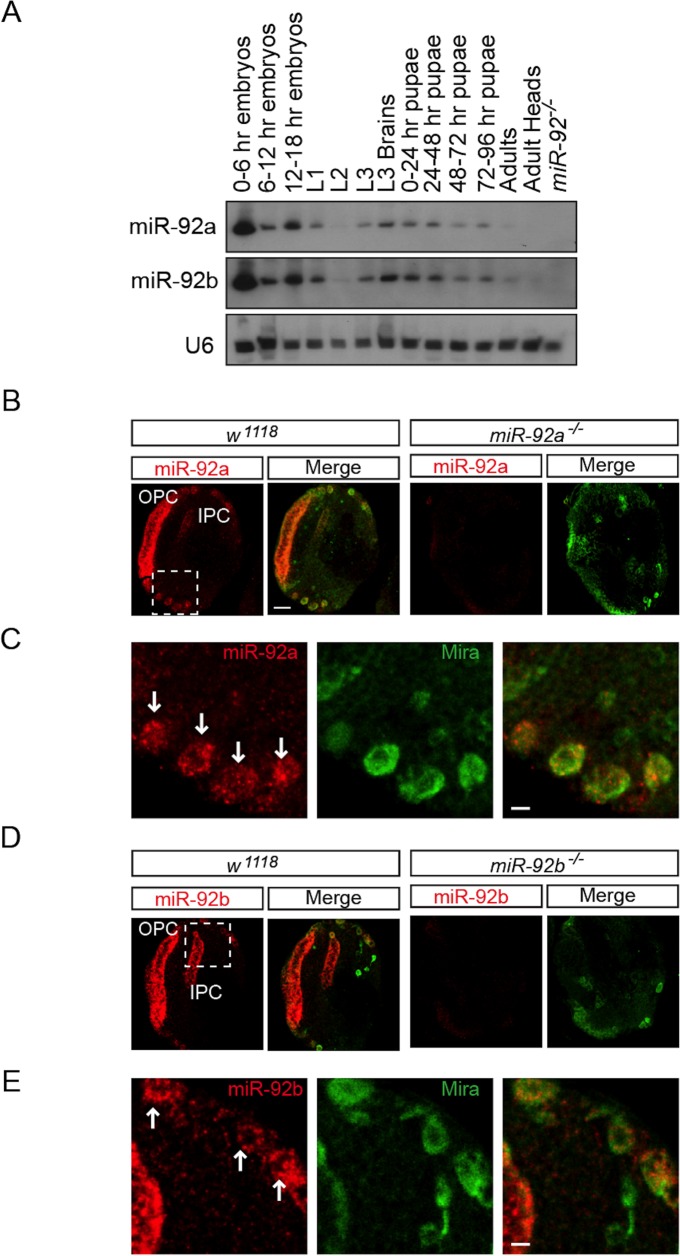
Expression profile of miR-92a and miR-92b in third Instar larval brain. (A) Northern blot analysis of total RNA isolated from staged wild type flies (lanes 1–13) and *miR-92* ^*–/–*^ mutant third instar larvae (lane 14). RNA was probed for miR-92a (top), miR-92b (middle), and U6 (bottom). (B) In situ analysis of mir-92a in the wild type and *miR-92a* ^*-/-*^ larval brains sectioned horizontally. Neuroblasts are labeled with Miranda (green). Scale bar: 20 μm. (C) Magnified view of boxed area in B. Arrows indicate neuroblasts. Scale bar: 5 μm. (D) In situ analysis of mir-92b in wild type and *miR-92b* ^*-/-*^ third instar larval brain horizontal sections. (E) Magnified view of boxed area in B. Arrows indicate neuroblasts. Scale bar: 5 μm. OPC: Outer proliferation center, IPC: Inner proliferation center.

The miR-92 family is evolutionarily conserved (S1A Fig) but its function in neural development in *Drosophila* is unknown. In *Drosophila*, the miR-92 family consists of miR-92a and miR-92b, which have the same seed sequence (miRbase, S1A Fig). We first characterized the expression of miR-92a and miR-92b in *Drosophila* at different stages of development by northern blot analysis and miRNA Taqman assay. Both miRNAs were expressed at high levels during the embryonic, larval, and pupal stages and at relatively low levels in adult flies; during the third instar larval stage, expression was enriched in the brain (Fig 1A and S1B Fig). miR-92a expression level is mostly higher than that of miR-92b at different developmental stages (S1B Fig).

**Fig 2:**
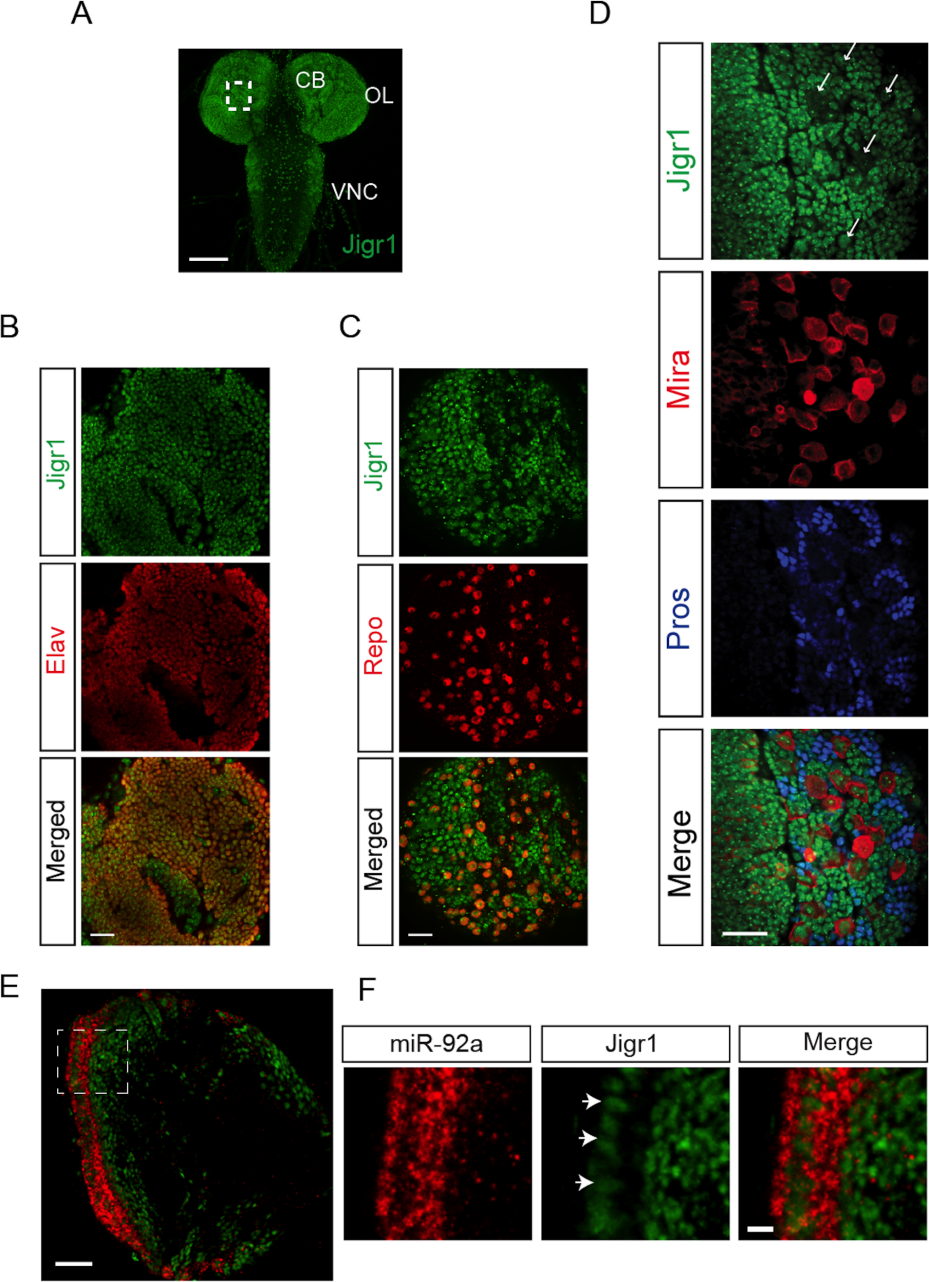
Expression profile of Jigr1 in third instar larval brain. (A) Jigr1 expression (green) in wild type third instar larval brain. Z-projection of optical sections is shown. Scale bar: 100 μm. CB: Central Brain, OL: Optic lobe, VNC: Ventral Nerve Cord. (B) Jigr1 expression (green) in post mitotic neurons labeled with Elav (red). Scale bar: 20 μm (C) Jigr1 expression (green) in glial cells labeled with glial specific transcription factor, Repo, (red) (D) High-magnification images of boxed area in A. Neuroblasts are labeled with Miranda (Mira, red), and ganglion mother cells (GMCs) and neurons are immunostained with Prospero (Pros, blue). Single optical section is shown. Arrows indicate neuroblasts. Scale bar: 20 μm. (E) Horizontal sections of third instar larval brains are labeled with miR-92a (red) and Jigr1 (green). Dashed lines outlines neuroepthelial cells (NE). Scale Bar: 20 μm. (F) Magnified view of dashed box in 2E. Arrows indicate miR-92a positive cells. Scale Bar: 5 μm.

To determine which cell types express miR-92a and miR-92b, we analyzed third instar larval brains by RNA *in situ* hybridization. Both miR-92a and miR-92b were expressed in the optic lobe and central brain in wild type flies; this expression was absent in *miR-92a* ^*–/–*^ and *miR-92b* ^*–/–*^ flies (please see detailed generation and characterization of these mutant flies below), respectively (Fig 1B–1E, S2B and S2C Fig). In the optic lobe, miR-92a co-expressed with Discs large (Dlg) in neuroepithelial cells [14] (S2A Fig). In the central brain, miR-92a and miR-92b preferentially co-expressed with the neuroblast markers Miranda (Mira) (Fig 1B–1E) and Deadpan (Dpn) (S2B Fig and Fig 2C). These results raise the possibility that miR-92 family may contribute to neuroblast development.

### Jigr1 and miR-92 Have Complementary Expression Patterns

Based on the FlyBase, *miR-92a* and *miR-92b* are located on the right arm of chromosome 3; *miR-92a* resides in the first intron of *jigr1*, and *miR-92b* resides downstream of *jigr1* coding region. Since miR-92a is in the intron of *jigr1*, we asked whether *jigr1* is also expressed in neural progenitor cells in larval brain. To assess the expression pattern of *jigr1*, we generated an antibody specific to first 110 amino acids of Jigr1. Nuclear Jigr1 signal was detected in the third instar larval brain (Fig 2A). High *jigr1* expression was present in neurons and glial cells (Fig 2B and 2C). Unexpectedly, we found that *jigr1* is expressed at low levels in neuroepithelial cells and neuroblasts, even though it is highly expressed throughout the nervous system (Fig 2D and 2E). Double labeling of miR-92a and Jigr1 in third instar larval brains confirms their complementary expression pattern (Fig 2E and 2F).

### *Drosophila* miR-92a and miR-92b and Their Host Gene *jigr1* Are Expressed in the Same Transcriptional Unit in Larval Brain

**Fig 3:**
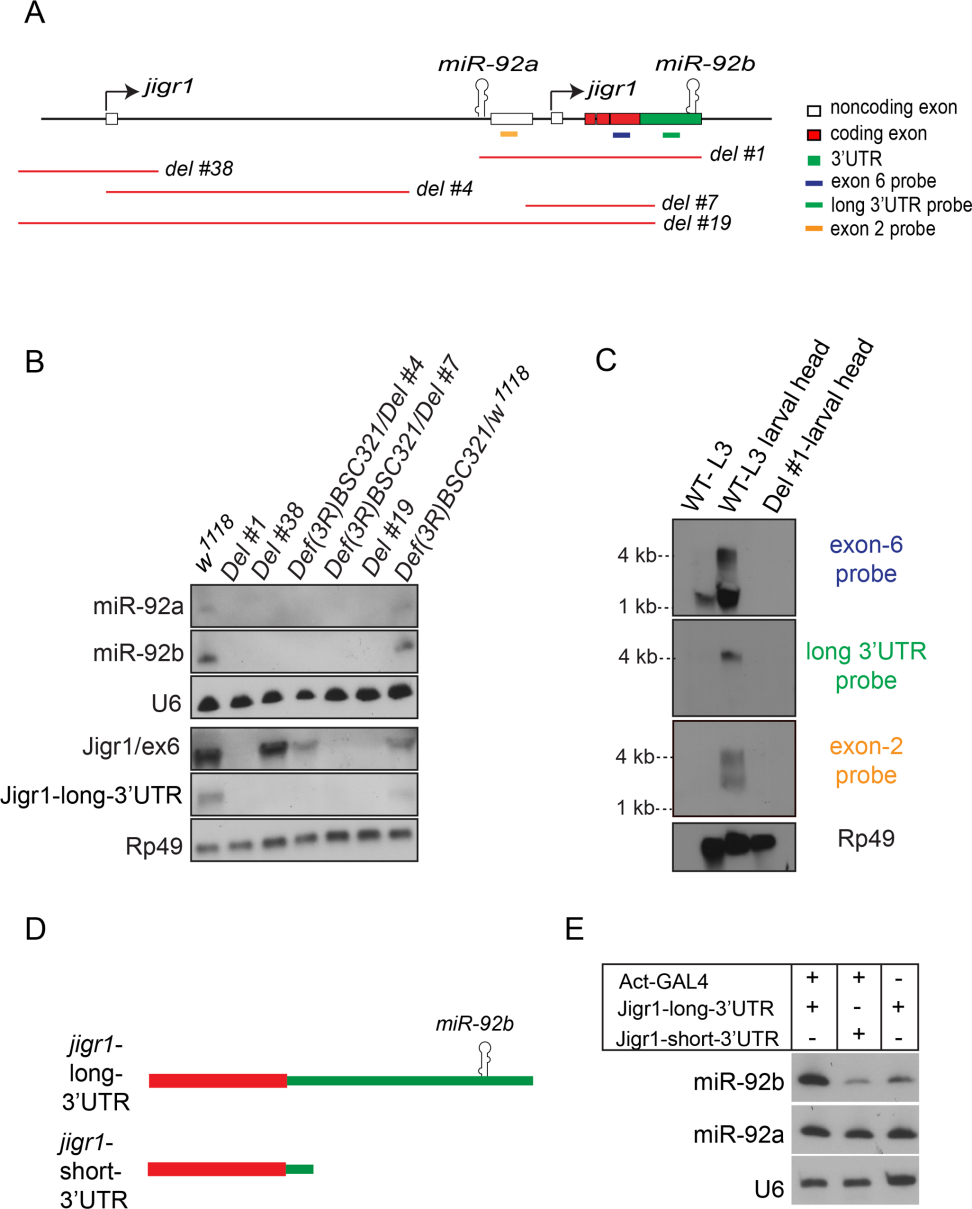
*miR-92a* and *miR-92b* are transcribed in the same transcriptional unit. (A) Schematic representation of the genomic arrangement of *miR-92a*, *miR-92b* and *jigr1* based on results from this study. Red lines indicate deleted regions. (B) Northern blot analysis of total RNA extracted from third instar larval heads of the indicated genotypes. RNA was probed for miR-92a, miR-92b, U6, Jigr1 (exon 6 probe), Jigr1 (long 3’UTR probe) and rp49. Deletion lines, *Del #4* and *Del #7*, are homozygous lethal but in trans to the bigger deficiency covering the locus, *Def(3R)BSC321*, they are viable and used like this for the analysis. (C) Northern blot analysis of RNA from wild type whole third instar larvae (lane 1), wild type larval heads (lane 2) and *Del #1* (lane 3) third instar larval heads. Probes specific for all the isoforms of *jigr1* (exon 6 probe, first panel), for the long 3’UTR (second panel), and for alternatively spliced noncoding exon 2 (third panel), and rp49 (bottom panel) were used. (D) Schematic representation of *jigr1* constructs. (E) Northern blot analysis of total RNA from HEK 293T cells transfected with UAST-*jigr1*-long-3’UTR or UAST-*jigr1*-short-3’UTR plasmids together with the Actin-Gal4 plasmid. Cells transfected with UAST-*jigr1*-long-3’UTR alone served as negative controls. miR-92b, miR-92a, and control U6 probes were used. The miR-92b probe recognizes endogenous miR-92b in HEK 293T cells.

miR-92a and miR-92b have the same expression profile (Fig 1 and S2 Fig), which suggests that they may be co-transcribed. To investigate this possibility, we generated a series of deletions at the *jigr1* locus by FLP-FRT [15] or cre/loxP deletions (Fig 3A and S3A Fig) and analyzed expression of miR-92a and miR-92b by northern blot (Fig 3B). If *miR-92a* and *miR-92b* were transcribed together, deleting the promoter region of *miR-92a* would prevent expression of both miRNAs. Indeed, in *Del #38* and *Del #4* flies, which lack the sequence upstream of *miR-92a*, expression of both miR-92a and miR-92b was lost (Fig 3B). Consistent with this, miR-92b expression was also lost in *Del #19* flies, which lack *miR-92a* and the coding region of *jigr1* (Fig 3B). Deleting only the coding region of *jigr1* in *Del #7* also eliminates both miR-92a and miR-92b expression (Fig 3B). Although *Del #38* and *Del #4* are 12.5 kb and 6.6 kb upstream of *miR-92b*, respectively, the loss of miR-92b expression resulting from these deletions suggests that a single long transcript contains *miR-92a* and *miR-92b*.

To determine whether *miR-92a* and *miR-92b* are co-expressed with *jigr1*, we analyzed *jigr1* expression by northern blot in wild type and deletion lines. In wild type third instar larva brains, a probe specific for *jigr1* exon 6, which is found in all the isoforms, identified *jigr1* transcripts of 1.5 kb and 4 kb (Fig 3C). Surprisingly, 1.5 kb *jigr1* transcript can still be detected in *Del #38* and *Del #4* flies while the 4 kb transcript was lost (Fig 3B). Since these deletions only remove the first non-coding exon, the short isoforms of *jigr1* probably have an alternative promoter downstream of these deletions, as predicted in FlyBase. These results suggest that *miR-92a* and *miR-92b* share the same transcriptional unit with a *jigr1* isoform that hasn’t been previously annotated. Our results also suggest that *miR-92b* does not have its own promoter and is not intergenic but is located in the 3’UTR of a longer *jigr1* transcript in the *Drosophila* larval brain.

To confirm this finding, we used 3’ rapid amplification of cDNA ends (3’RACE) to map the 3’UTR of *jigr1*. We observed two *jigr1* 3’UTRs of different lengths. One is 203 bp long and the other is 1827 bp long and contains the *miR-92b* stem loop (S3B and S3C Fig). The presence of the neurally enriched long 3’UTR was also confirmed by RNA-seq analysis as reported in the FlyBase (S3D Fig). Next, we analyzed RNA from whole larvae and larval heads by northern blot. A long 3’UTR-specific probe identified only the 4-kb transcript (Fig 3C). The upper 4 kb transcript was fainter in whole larvae than in larval heads, indicating that it is more abundant in the brain (Fig 3C). We also identified another brain-enriched *jigr1* isoform in which intron 2 is retained in the transcript. A probe specific for this noncoding exon 2 recognized transcripts of 2.5 kb and 4 kb in larval brain (Fig 3C). Next, we asked whether the long *jigr1* transcript could give rise to miR-92b. In HEK 293T cells, expression of this transcript containing the miR-92b stem loop (Fig 3D) increased the amount of mature miR-92b, confirming that miR-92b can be processed from the 3’UTR of long *jigr1* transcript (Fig 3E).

### Generation of *miR-92a* and *miR-92b* Single- and Double-Knockout Mutant Flies

**Fig 4:**
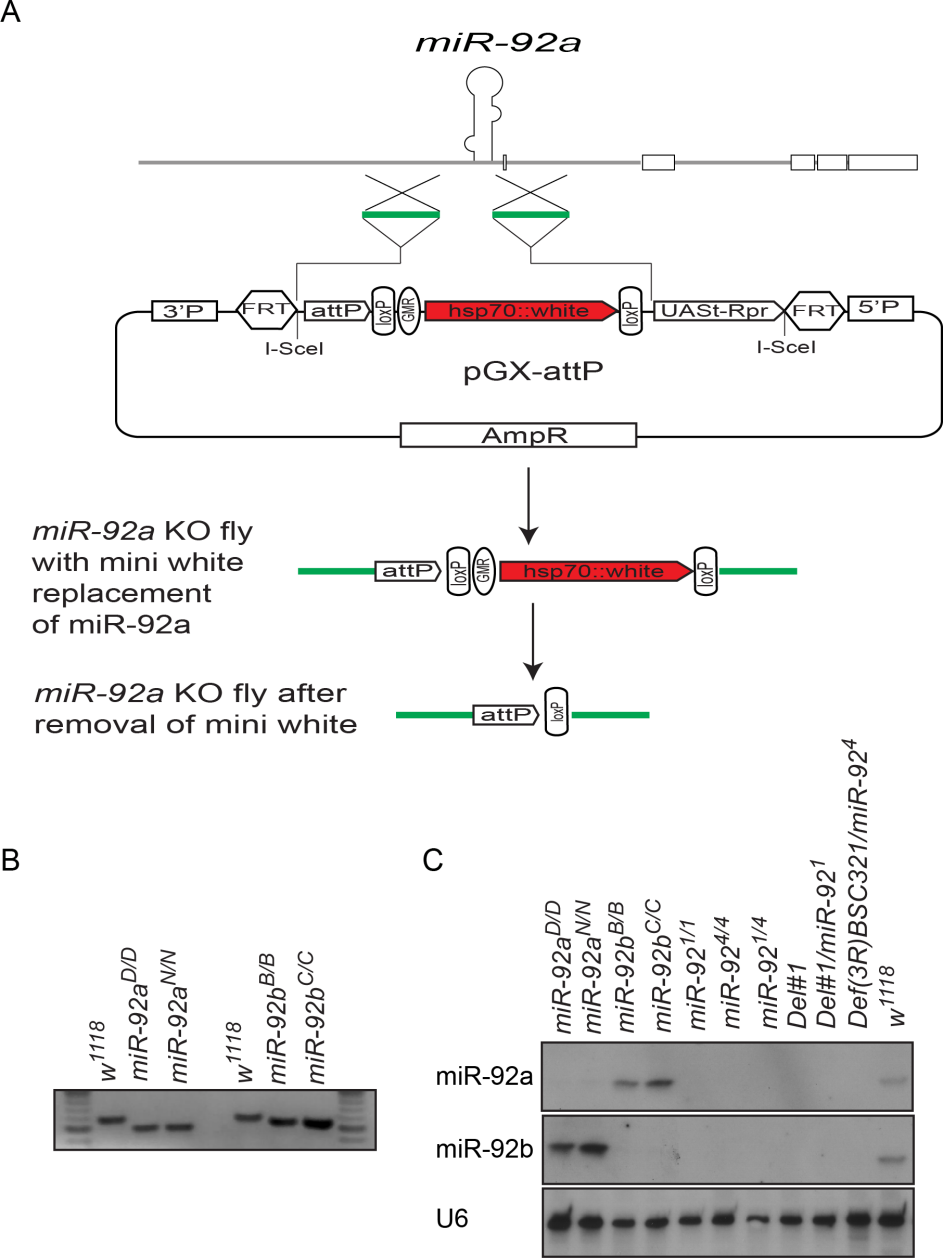
Generation of *miR-92a* ^*–/–*^ and *miR-92b* ^*–/–*^ flies by ends-out gene targeting. (A) Schematic of ends-out gene targeting strategy (modified from Huang et al., 2009). (B) DNA gel electrophoresis of PCR-amplified miR-92a (left) and miR-92b (right) locus from genomic DNA of wild type and *miR-92a* ^*–/–*^ and *miR-92b* ^*–/–*^ single knockout (KO) flies. (C) Northern blot analysis of miR-92a, miR-92b, and U6 expression in wild type, *miR-92a* ^*–/–*^, *miR-92b* ^*–/–*^, and *miR-92* ^*–/–*^ mutant third instar larvae.

To analyze the function of the miR-92 family in neurogenesis in *Drosophila*, we first generated *miR-92a* and *miR-92b* single mutants by ends-out homologous recombination in which 140 bp deletions were generated after mini white was removed by Cre/LoxP-mediated recombination (Fig 4A) [16]. Loss of miR-92a or miR-92b was confirmed by PCR (Fig 4B) and northern blot analysis (Fig 4C). In northern blot, miR-92a level seems to be higher in *miR-92b* ^*-/-*^ mutants and vice versa (Fig 4C). In order to obtain more quantitative data, we performed miRNA Taqman assay. In *miR-92a* ^*-/-*^ L3 heads, miR-92b expression was increased by 30% and in *miR-92b* ^*-/-*^ L3 heads, miR-92a expression was increased by 40% (S1B Fig).

Both *miR-92a* ^*–/–*^ and *miR-92b* ^–/–^ flies were viable and fertile and had no obvious phenotype, in contrast to a recent report showing *miR-92b* deletion mutant flies are larval lethal, in which both *miR-92b* and the *jigr1* coding region are deleted [17]. However, our flies homozygous for *Del #1*, in which both miR-92a/miR-92b and Jigr1 are deleted, are still adult viable. Thus, it is unclear what causes the lethality phenotype in *miR-92b* mutant flies generated by Chen et al [17]. Since miR-92a and miR-92b have an identical seed sequence and are similarly expressed, they might compensate for each other. So, using the same ends-out gene targeting method [16], we targeted the *miR-92b* locus on the *miR-92a* ^*–/–*^ mutant background to generate flies lacking both *miR-92a* and *miR-92b* (named as *miR-92* ^*–/–*^). Two independent lines, *miR-92* ^*4*^ and *miR-92* ^*1*^ were further characterized and used in this study. Unlike the single-knockout flies, which have a normal life span, *miR-92* ^*–/–*^ flies have a reduced life span; most die within a week (S4 Fig). Expression of miR-92a or miR-92b in the *miR-92* ^*–/–*^ background with *Insc-GAL4* that drives gene expression in neuroblasts largely rescued the life span defect, confirming that this phenotype is indeed due to loss of miR-92a and miR-92b (S4 Fig).

### miR-92a and miR-92b Maintain Neuroblast Self-Renewal by Inhibiting Premature Differentiation

**Fig 5:**
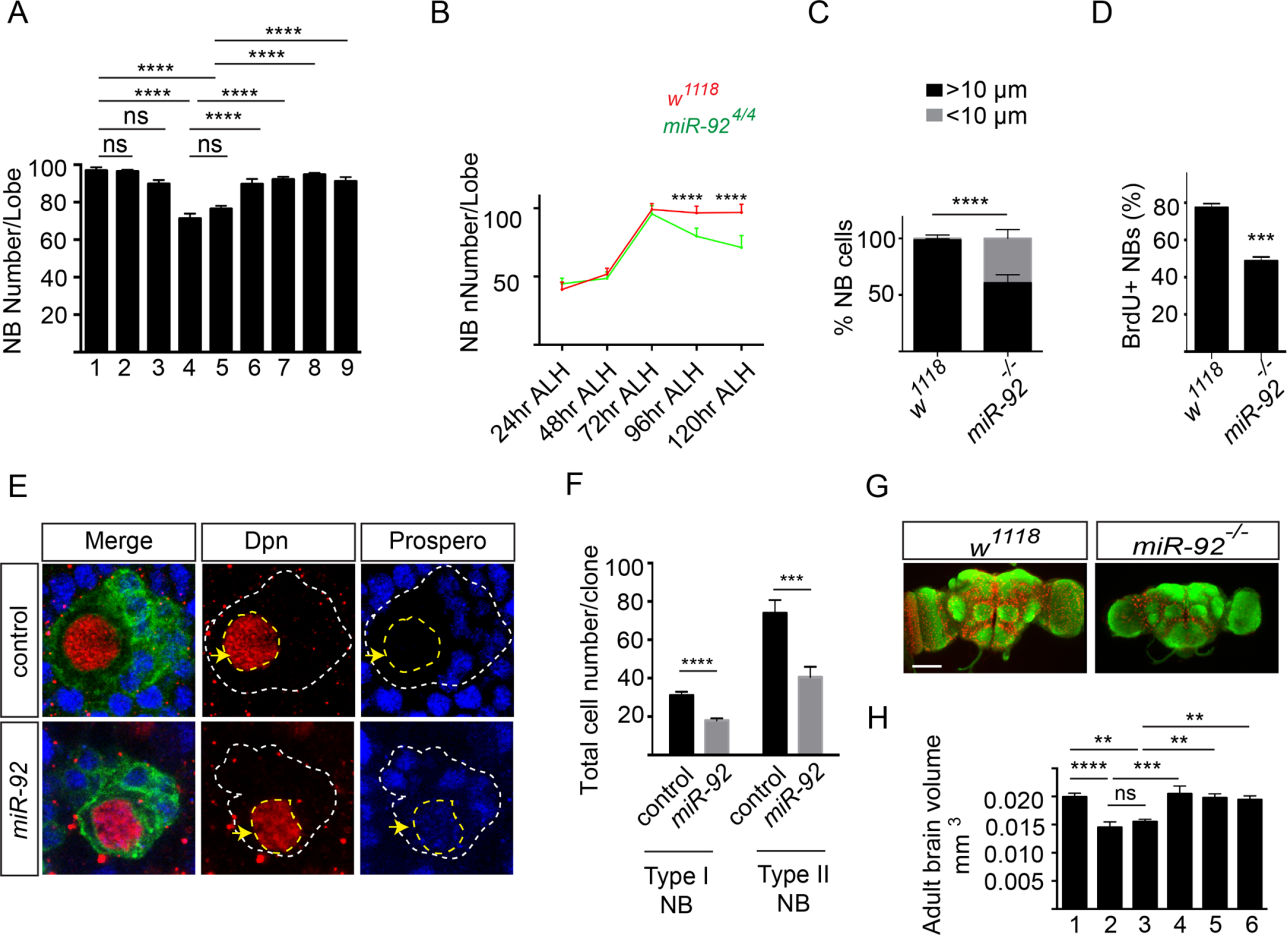
miR-92a and miR-92b regulate neuroblast self-renewal by inhibiting premature differentiation. (A) Number of brain neuroblasts at 120 hr ALH. 1: *w* ^*1118*^ (n = 17); 2: *miR-92a* ^*-/-*^ (n = 13); 3: *miR-92b* ^*-/-*^ (n = 12); 4: *miR-92* ^*4/4*^ (n = 15); 5: *miR-92* ^*1/4*^ (n = 20); 6: *Insc-GAL4/UAS-miR-92a; miR-92* ^*4/4*^ (n = 8); 7: *Insc-GAL4/UAS-miR-92b; miR-92* ^*4/4*^ (n = 8); 8: *Insc-GAL4/UAS-miR-92a; miR-92* ^*1/4*^ (n = 16); 9: *Insc-GAL4/UAS-miR-92b; miR-92* ^*1/4*^ (n = 15). Statistical significance was determined by one-way ANOVA. (B) Number of neuroblasts in wildtype (red) and *miR-92* ^*4/4*^ (green) mutants from 24 to 120 hr ALH. Statistical significance was determined by Student’s *t* test. Error bars show S.D. (C) Percentage of neuroblasts with diameter > 10 μm (black bar) and < 10 μm (grey bar) in wild type (n = 60) and *miR-92* ^*–/–*^ (n = 60) mutant brains at 96 hr ALH. Fisher’s exact test was used for statistical analysis. (D) Percentage of BrdU^*+*^ wild type control (n = 15) and *miR-92* ^*–/–*^ mutant (n = 16) larval neuroblasts. Statistical significance was determined by Student’s *t* test. (E) Neuroblast clones of wild type control and *miR-92* ^*4*^ mutants. Clones are marked with CD8::GFP (green). Yellow arrows mark Dpn^*+*^ neuroblasts. White dashed lines indicate the position of clones and yellow dashed line encircles the nucleus of neuroblast cell. Single focal planes are shown. (F) Comparison of wild type control (type I; n = 57, type II; n = 27) and *miR-92* ^*-/-*^ mutant (type I; n = 67, type II; n = 33) clone sizes. (G) Whole-mount image of wild type and *miR-92* ^*–/–*^ mutant male adult brains immunostained with the anti-HRP (green) to label axonal membranes and anti-Repo (red) to label glial cell nuclei. Scale bar: 100 μm. (H) Adult brain volume measured using ImageJ. 1: *w* ^*1118*^ (n = 10); 2: *miR-92* ^*4/4*^ (n = 10); 3: *miR-92* ^*1/4*^ (n = 8); 4: *Insc-GAL4/UAS-miR-92a; miR-92* ^*4/4*^ (n = 7); 5: *Insc-GAL4/UAS-miR-92a; miR-92* ^*1/4*^ (n = 9); 6: *Insc-GAL4/UAS-miR-92b; miR-92* ^*1/4*^ (n = 10). Statistical significance was determined by one-way ANOVA. Values are mean ± s.e.m. in all graphs unless otherwise stated. *: p < 0.05, **: p < 0.005, ***: p < 0.001, ****: p < 0.0001.

Since a high level expression of miR-92a and miR-92b is seen in neuroblasts, we examined larval brain neuroblasts in *miR-92* ^*–/–*^ flies (S5A Fig). At 96 and 120 hr after larval hatching (ALH), there were about 100 neuroblasts in the central brain in wild type, *miR-92a* ^*–/–*^, and *miR-92b* ^*–/–*^ flies, as shown by staining with the neuroblast marker Dpn (Fig 5A, S5A and S5B Fig). However, *miR-92* ^*–/–*^ flies had significantly fewer neuroblasts (Fig 5A and S5B Fig). At 120 hr ALH, the reduction was ~23% in *miR-92* ^*1/4*^ flies (p < 0.005) and ~30% in *miR-92* ^*4/4*^ flies (p < 0.0001) (Fig 5A). These reductions were fully rescued by expression of either miR-92a or miR-92b with *Insc-GAL4*, suggesting miR-92a and miR-92b are required cell autonomously in neuroblasts (Fig 5A and S5B Fig).

The decreased number of neuroblasts in *miR-92* ^*–/–*^ flies could reflect defects in neuroblast formation, cell survival or self-renewal. To distinguish between these possibilities, we first quantified neuroblasts at earlier stages of development. At 24, 48 and 72 hr ALH, wild type and *miR-92* ^*–/–*^ flies had equal numbers of neuroblasts, suggesting that the phenotype is not due to defect in neuroblast formation or failure of neuroblasts to re-enter the cell cycle (Fig 5B). After 72 hr ALH, the number of neuroblasts in wild type did not change, as they are mainly undergoing asymmetric cell division. However, in *miR-92* ^*–/–*^ mutants, neuroblasts were lost progressively, resulting in 18% decrease at 96 hr ALH and a further 30% decrease at 120 hr ALH (p < 0.0001) (Fig 5B).

To determine whether neuroblast survival is defective in *miR-92* ^*–/–*^ flies, we immunostained the cells for activated Caspase-3. No significant increase in caspase activation was detected (S5C Fig), and blocking apoptosis by expressing the anti-apoptotic protein P35 in neuroblasts did not rescue this phenotype (S5D Fig). In larval brain, neuroblasts maintain their population by asymmetric cell division, so a defect in this process might also lead to loss of neuroblasts, if these cells generated two differentiated daughter cells instead of self-renewing. Neither the apical localization of aPKC nor the basal localization of Numb or Miranda was disrupted in *miR-92* ^*–/–*^ neuroblasts (n = 30) (S6A and S6B Fig). In addition to the differential segregation of cell fate determinants, asymmetric cell division also generates unequal sized daughters and these two mechanisms are independently regulated [18]. In order to determine if size asymmetric division is disrupted in *miR-92* ^*-/-*^ mutants, we have stained the third instar larval brains with Miranda and phosphor-histone H3. We didn’t observe any size symmetric neuroblast division in *mir-92* ^*-/-*^ mutant neuroblasts (n = 33) (S6C Fig). Thus, defective asymmetric cell division is unlikely to be the mechanism of neuroblast loss in *miR-92* ^*–/–*^ larvae.

Another possibility that could result in loss of neuroblasts is premature differentiation of neuroblasts, a process that correlates with a reduction in cell size [19, 20]. Therefore, we measured neuroblast diameter in control and *miR-92* ^*–/–*^ brains. In controls, almost all neuroblasts were >10 μm in diameter at 96 hr ALH; however, in *miR-92* ^*–/–*^ mutants, 39% of neuroblasts had a reduced cell diameter (p < 0.0001) (Fig 5C). To determine whether *miR-92* ^*–/–*^ neuroblasts were still capable of dividing, we performed a BrdU incorporation assay. Almost 80% of wild type neuroblasts incorporated BrdU, versus only 50% of *miR-92* ^*–/—*^neuroblasts, suggesting that loss of miR-92a and miR-92b causes premature exit from the cell cycle (Fig 5D).

Next, we analyzed *miR-92* ^*–/–*^ brains for expression of Prospero, a homeodomain transcription factor that is localized in the cortex in self-renewing larval neuroblasts but transiently moves to the nucleus in quiescent or terminally differentiating neuroblasts [21,22]. In 3–5% neuroblasts in *miR-92* ^*–/–*^ brains, Prospero was localized in the nucleus and decreased the expression level of the neuroblast nuclear marker Dpn (S5E Fig). Next we analyzed *miR-92* phenotype using mosaic analysis of repressible cell marker (MARCM) method [23]. Wild type clones contained single Dpn^+^, Pros^-^ neuroblast cell surrounded by several Dpn^-^, Pros^+^ daughter cells. In *miR-92* mutant clones, some Dpn^+^ neuroblast cells also showed nuclear Prospero expression (Fig 5E and S6D Fig). Moreover, the size of *miR-92* mutant clones was significantly reduced (Fig 5F). In BrdU chase experiment in MARCM clones, around 40% of the wild type neurons were BrdU^+^ while only 23% of *miR-92* ^*–/–*^ neurons were BrdU^+^. Thus, control of neuroblast self-renewal by miR-92a and miR-92b is achieved by preventing their early differentiation. Indeed, although no obvious difference in body size was observed for adult flies, the size of the ventral nerve cord of *miR-92* ^*–/–*^ adult flies is 15% smaller (p < 0.05), the brains were also smaller than those of control flies (Fig 5G and 5H), which is probably due to reduced proliferation (Fig 5D) and premature differentiation (Fig 5E) of neuroblasts that lead to reduced production of neurons and glial cells.

### Some *jigr1* Isoforms Are Direct Targets of miR-92a and miR-92b In Vivo

**Fig 6:**
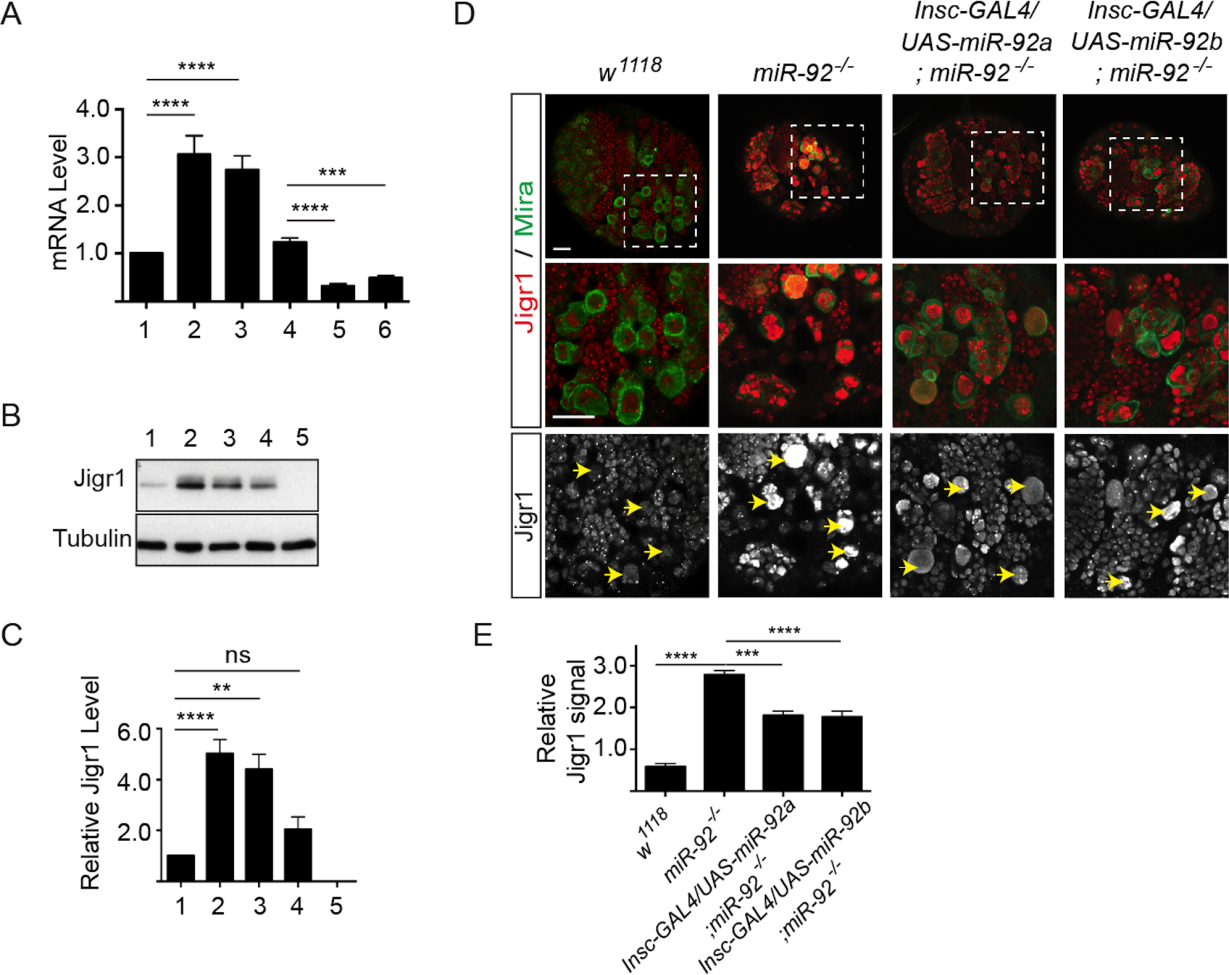
Elevated Jigr1 levels in *miR-92* ^*-/-*^ brains. (A) qRT-PCR analysis of relative expression of *jigr1* in third instar larval brains. 1: *w* ^*1118*^; 2: *miR-92* ^*4/4*^; 3: *miR-92* ^*1/4*^. 4: *Pros-Gal4/+*, 5: *Pros-Gal4/UAS-miR-92a*; 6: *Pros-Gal4/UAS-miR-92b*. (B) Western blot analysis of Jigr1 protein in lysates of third instar larval brain. 1: *w* ^*1118*^; 2: *miR-92* ^*4/4*^; 3: *miR-92* ^*1/4*^; 4: *Def(3R)BSC321/miR-92* ^*4*^; 5: *Del #1*. (C) Expression level of Jigr1 normalized to alpha-Tubulin. Western blots are representative of three independent experiments. 1: *w* ^*1118*^; 2: *miR-92* ^*4/4*^; 3: *miR-92* ^*1/4*^; 4: *Def(3R)BSC321/miR-92* ^*4*^; 5: *Del #1*. (D) Single confocal sections of third instar larval brains of the indicated genotypes stained for Jigr1 (red) and Miranda (green). High-magnification images of boxed areas in top panel are shown in the middle panel. Arrowheads indicate neuroblasts. Scale bar: 20 μm. (E) Jigr1 level in neuroblasts relative to that in neighboring ganglion mother cells. Ten larval brains of each genotype were analyzed One-way ANOVA was used for statistical comparisons unless otherwise stated. Values are mean ± s.e.m. in all graphs. *: p < 0.05, **: p < 0.005, ***: p < 0.001, ****: p < 0.0001.

miRNAs regulate the expression of their target genes post-transcriptionally, mostly by binding to target sequences in the 3’UTR and mediating mRNA degradation and/or translation inhibition [24]. We were intrigued by the finding that *miR-92* family and *jigr1* show complementary expression pattern in the larval brain (Fig 2E), which raises the possibility that *jigr1* mRNA might be a target of miR-92 family. Indeed, intragenic miRNAs may target their own host genes and function as negative feedback regulators [25–30]. To determine whether *jigr1* is a functional target of miR-92 in vivo, we first analyzed *jigr1* mRNA levels in control and *miR-92* ^*–/–*^ third instar larval brains by qRT-PCR. *jigr1* transcripts were almost threefold more abundant in *miR-92* ^*–/–*^ brains (p < 0.0001) (Fig 6A). Moreover, overexpression of miR-92a or miR-92b by *Pros-Gal4* in wild type background decreased *jigr1* level in third instar larval brain (Fig 6A and S7A Fig). Analysis of Jigr1 expression in third instar larval brains by western blot revealed a single band of ~36 kDa that is absent in *Del #1*, in which the entire *jigr1* coding sequence is deleted, confirming the specificity of the Jigr1 antibody. Consistent with the increase in transcript levels, Jigr1 protein was also substantially more abundant in *miR-92* ^*–/–*^ larval brains (Fig 6B and 6C).

Although miR-92a and miR-92b are transcribed together with the long *jigr1* transcript, miR-92a and miR-92b are enriched in neuroblasts, which express Jigr1 at a low level. However, in *miR-92* ^*–/–*^ brains, Jigr1 is expressed at high levels, especially in neuroblasts (Fig 6D). Since all *miR-92* ^*–/–*^ phenotypes in neuroblasts can be rescued by expression of miR-92a or miR-92b, we wanted to see if the elevated Jigr1 level could be suppressed too. Indeed, expression of miR-92a or miR-92b in *miR-92* ^*–/–*^ mutants significantly reduced *jigr1* expression in neuroblasts (Fig 6D and 6E). These results suggest that miR-92 directly regulates its host gene *jigr1*.

**Fig 7:**
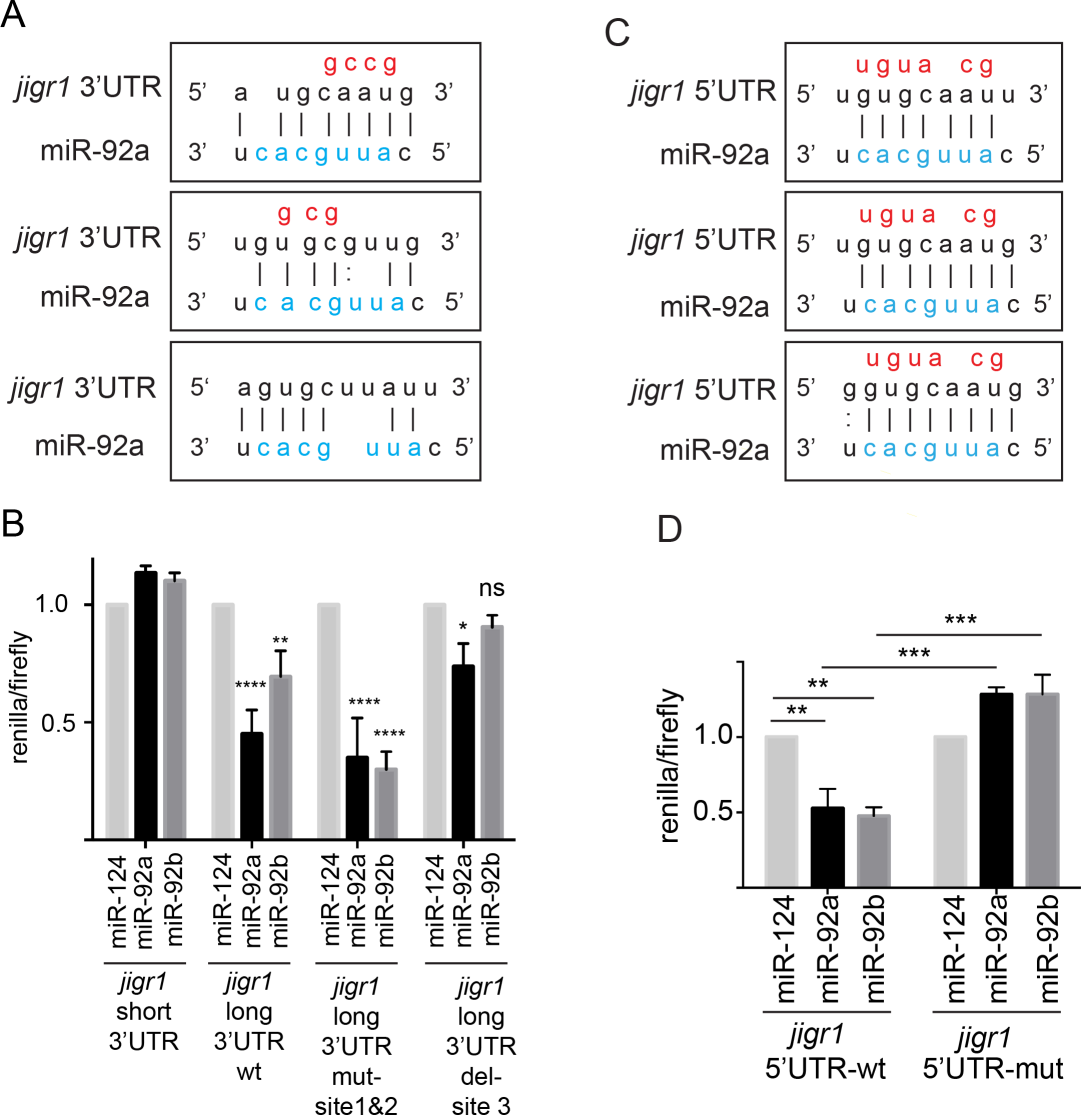
miR-92a and miR-92b target their host gene *jigr1*. (A) Predicted miR-92a and miR-92b binding sites in the 3’UTR of *jigr1*. The seed sequences of miR-92a and miR-92b are the same and shown in blue and mutated nucleotides are shown in red. (B) Dual luciferase assay of HEK 293T cell lysates cotransfected with miR-92a, miR-92b or miR-124 expression vectors together with dual luciferase construct containing the wild type or mutant 3’UTR of *jigr1* at 3’ of Renilla luciferase. Bar graph shows normalized mean luciferase activity of cells transfected with miR-92 expression plasmid to that of cells transfected with control miRNA from three independent experiments. Statistical significance was determined by two-way ANOVA. (C) Predicted miR-92a and miR-92b binding sites at the 5’UTR of *jigr1*. Mutated nucleotides are shown in red. (D) Dual luciferase assay of lysates of HEK 293T cells co-transfected with miR-92a, miR-92b, or control miRNA (miR-124) expression vectors together with a firefly luciferase construct containing the wild type or mutant 5’UTR of *jigr1*. Bar graph shows normalized mean values of the luciferase activity of cells transfected with a miR-92 expression plasmid relative to that of cells transfected with control miRNA from three independent experiments. Statistical significance was determined by two-way ANOVA. Values are mean ± s.e.m. in all graphs. *: p < 0.05, **: p < 0.005, ***: p < 0.001, ****: p < 0.0001.

To further confirm that *jigr1* is a direct target of miR-92a and miR-92b, we used a reporter silencing assay. The short 3’UTR of *jigr1* has no binding site for miR-92a or miR-92b, while the long 3’UTR has three predicted miR-92 binding sites (Fig 7A). In HEK 293T cells, co-expression of miR-92a and/or miR-92b with a luciferase reporter carrying the *jigr1* long 3’UTR in HEK 293T cells significantly suppressed luciferase activity (Fig 7B). Mutating the first two predicted binding sites did not abolish the suppression of luciferase activity by miR-92a and/or miR-92b. However, deletion of site 3 eliminated the suppression, showing that *jigr1* mRNA is a direct target of miR-92a and miR-92b (Fig 7B).

miRNAs can also regulate mRNAs by binding to the 5’UTR [31]. *Jigr1* mRNA has three potential miR-92 binding sites in the alternatively spliced noncoding exon 2 (Figs 3A and 7C). We cloned the sequence containing the intact and the mutated binding sites to the 5’UTR of the luciferase gene and co-transfected it together with miR-92a and/or miR-92b and control miRNA. Overexpression of miR-92a or miR-92b suppressed luciferase activity from a reporter with intact binding sites but had no effect on a reporter containing mutated binding sites (Fig 7D). Thus, *jigr1* isoforms containing this 5’UTR can be regulated directly by miR-92a and miR-92b through its 5’UTR.

As we showed previously (Fig 5D and S5C Fig), some *miR-92* ^*-/-*^ mutant neuroblasts have nuclear Prospero expression. Prospero is also a predicted target of *miR-92a* and *miR-92b* (Targetscan). In order to investigate whether *prospero* mRNA can be directly downregulated by *miR-92a* and *miR-92b*, we performed luciferase reporter assay. *Prospero* mRNA has six isoforms and three alternative 3’UTRs with varying lengths (Flybase). Two short 3’UTRs contain one miR-92a/miR-92b binding site and the long 3’UTR contains three. Luciferase activity was measured in HEK 293T cells transfected either with *miR-92a*, *miR-92b* or empty vector together with psicheck2-pros-short-UTR or psicheck2-pros-long-UTR plasmids. We couldn’t detect any significant downregulation of luciferase activity by either *miR-92a* or *miR-92b*. Thus, *prospero* mRNA is not a direct target of miR-92a and miR-92b (S7B and S7C Fig).

### *Jigr1* Upregulation Leads to Premature Neuroblast Differentiation in *miR-92* ^*–/–*^ Flies

**Fig 8:**
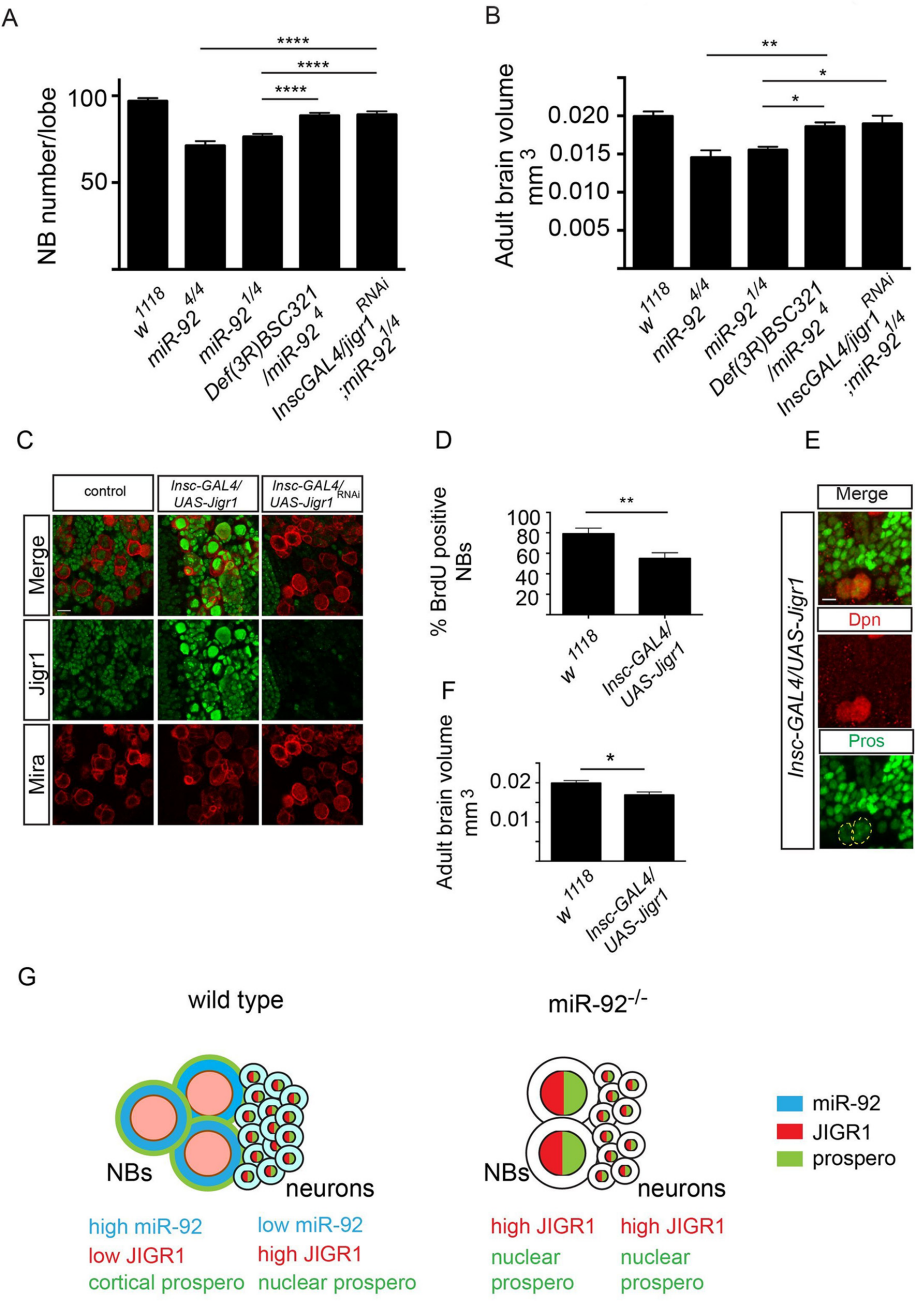
*Jigr1* upregulation is responsible for premature neuroblast differentiation in *miR-92* ^*–/–*^ flies. (A) Neuroblast number in third instar larval brains of the indicated genotypes at 120 hr ALH. *w* ^*1118*^ (n = 17); *miR-92* ^*4/4*^ (n = 15); *miR-92* ^*1/4*^ (n = 20); *Def(3R)BSC321/miR-92* ^*4*^ (n = 17); *Insc-GAL4/UAS-Jigr1* ^*RNAi*^ *; miR-92* ^*1/4*^ (n = 14). (B) The size of Adult brains of indicated genotypes. *w* ^*1118*^ (n = 10); *miR-92* ^*4/4*^ (n = 10); *miR-92* ^*1/4*^ (n = 8); *Def(3R)BSC321/miR-92* ^*4*^ (n = 12); *Insc-GAL4/UAS-Jigr1* ^*RNAi*^ *; miR-92* ^*1/4*^ (n = 8). (C) Immunostaining of third instar larval brains of wild type (left panel), *Insc-GAL4/UAS-jigr1* (middle panel) and *Insc-GAL4/UAS-jigr1-RNAi* (right panel) flies. Green: Jigr1. Red: Miranda. Scale Bar: 20 m. (D) Percentage of BrdU^*+*^ neuroblasts in wild type control (n = 12), *Insc-GAL4/UAS-jigr1* (n = 15) and *Insc-GAL4/UAS-jigr1-RNAi* (n = 9) larval brains. (E) Immunostaining of *Insc-GAL4/UAS-jigr1*brains with Prospero (green) and Dpn (red) at 120 hr ALH. (F) Adult brain volume of wild type control (n = 10) and *Insc-GAL4/UAS-jigr1* (n = 9) flies. (G) Working model summarizing the expression and the role of miR-92 in larval neuroblasts. Statistical significance was determined by One-Way ANOVA. Values are mean ± s.e.m. in all graphs. *: p < 0.05, **: p < 0.005, ***: p < 0.001, ****: p < 0.0001.

To determine whether upregulation of *jigr1*contributes to the *miR-92* mutant phenotype, we carried out genetic interaction experiments. If an elevated level of Jigr1 causes premature differentiation of neuroblasts in *miR-92* mutants, reducing Jigr1 levels in *miR-92* mutants could suppress this phenotype. Indeed, limiting the Jigr1 level, either with the deficiency covering the *jigr1* locus or by downregulating it in neuroblasts with *UAS-jigr1-RNAi*, largely rescued reduced neuroblast numbers in *miR-92* ^*–/–*^ flies (Fig 8A) and brain size (Fig 8B). Moreover, upregulation of *jigr1* also contributed significantly to the life span defect in *miR-92* ^*–/–*^ flies (S8 Fig). Thus, a key function of these miRNAs is to suppress the expression of its host gene in developing neuroblasts. Moreover, in order to assess whether jigr1 upregulation mimics the *miR-92* ^*–/–*^ phenotype, we overexpressed *jigr1* in larval brain neuroblasts (Fig 8C). We observed decreased BrdU uptake and premature localization of Prospero in larval neuroblasts. In addition overexpression of Jigr1 in neuroblasts led to a reduction in adult brain size (Fig 8F) suggesting that Jigr1 overexpression is sufficient to cause premature cell cycle exit and differentiation of neuroblasts (Fig 8D and 8E). Taken together, our results suggest that miR-92 family maintains neuroblast self-renewal in part through limiting Jigr1 expression.

## Discussion

In this study, we report an unusual genomic arrangement in which miR-92a and miR-92b are embedded in the intron and 3’UTR of the host gene *jigr1*, respectively. In neuroblasts, miR-92a and miR-92b were highly expressed as a single transcriptional unit also containing *jigr1* coding region. Genetic analysis in *Drosophila* showed that downregulation of *jigr1* by intragenic miR-92a and miR-92b is required for neuroblast self-renewal, providing an example of the functional significance of miRNA–host gene interactions in animal development.

Nearly half of the miRNAs in mammals and *Drosophila* are located within protein-coding genes [4, 25]. Most of these intragenic miRNAs are co-expressed with their host genes, but both positive and negative feedback regulation of host gene expression and function by miRNAs remains largely unknown[26–30, 32]. Most intragenic miRNAs are located in the introns of their host genes and are processed by the mirtron pathway, bypassing the microprocessor complex [33]. On the other hand, miRNAs are rarely located in the 3’UTR of a protein-coding gene, and the effect of this organization on host gene expression or miRNA processing is not clear. The exonic miR-198, which is located in the 3’UTR of the gene encoding human folistatin like 1, is processed from a single transcript with its host gene in a mutually exclusive way [34]. In contrast, direct regulation of some *jigr1* isoforms by miR-92a and miR-92b largely accounts for the observed complementary expression domains of *jigr1* and these miRNAs (Figs 3 and 6), although we cannot completely rule out the possibility other mechanisms may also contribute to jigr1 repression.

Through a genetic knockout of both *miR-92a* and *miR-92b* in *Drosophila*, which has not been done so far in any other model organism, we discovered a novel function for miR-92a and miR-92b in neuroblast self-renewal. Our findings are consistent with results obtained in mammals. However, unlike *Drosophila*, human and mouse *miR-92a* and *miR-92b* genes are not intragenic. Mouse miR-92a genes are located in two clusters: miR-17-92 and miR-106a-303. In developing mouse neocortex, the miR-17-92 cluster promotes neural stem cell expansion and regulates the transition to intermediate progenitors through repression of Pten by miR-19 and Tbr2 by miR-92a [35]. Similarly, acute loss and gain of miR-92b function in mouse cortex showed that miR-92b restricts the generation of intermediate progenitor cells by suppressing Tbr2 [36]. Moreover, miR-92b maintains asymmetric division of neural stem cells by restricting Tis21 expression in mouse neocortex [37]. However, phenotypes caused by loss of both miR-92a and miR-92b in mammals have not been reported yet. Here we found that miR-92a and miR-92b work in concert to restrict Jigr1 expression in *Drosophila* larval neuroblasts and thereby maintain the neuroblast pool.

Jigr1, a putative MADF-domain-containing transcription factor of unknown function expressed ubiquitously in the larval central nervous system, is expressed at low levels in neural progenitor cells. Our findings suggest that the progressive loss of neuroblasts in *miR-92* ^*–/–*^ brains is due to premature differentiation of these cells resulted from cell-autonomous effect of loss of miR-92, as shown by ectopic expression of nuclear Prospero, decreased BrdU uptake, and reduced cell size. Upregulation of Jigr1 seems to play a role in nuclear Prospero expression in *miR-92* ^*–/–*^ neuroblasts, since reducing Jigr1 expression eliminated this phenotype. Moreover, overexpression of Jigr1 in neuroblasts on a wild type background leads to ectopic Prospero expression and a premature differentiation phenotype. In summary, our results reveal a local regulatory loop in which miR-92a and miR-92b are expressed in the *jigr1* transcription unit and also work in concert to prevent premature differentiation of neuroblasts by limiting expression of the host gene.

## Materials and Methods

### Fly Stocks

Fly stocks were maintained at 25°C on standard medium. The following fly stocks were obtained from the Bloomington *Drosophila* Stock Center: *w* ^*1118*^, *Elav-GAL4*, *Def(3R)BSC321*, y[1] w[*]/Dp(2;Y)G, *P{hs-hid}Y; P{70FLP}11 P{70I-SceI}2B noc[Sco]/CyO*, *P{hs-hid}4*, y[1] w[67c23] P{y[+mDint2] = Crey}1b; D[*]/TM3, *Sb[1]*, *hs-flp70; Tub-Gal4 UAS-mCD8*:*GFP; FRT82B Tub-Gal80*, *FRT82B*. *UAS-jigr1-RNAi (#100970) was* obtained from the Vienna *Drosophila* Resource Center. *Insc-GAL4* and *Pros-GAL4* were kind gift of Chris Doe (University of Oregon).

### Generation of Transgenic Flies and Deletion Lines

The UAS-dsRed-miR-92a vector was from the *Drosophila* RNAi Screening Center, and UAS-miR-92b was generated by amplifying the 500–base pair pri-miR-92b from genomic DNA and cloning it into the pTW vector. Primers for PCR are listed in S1 Table. *UAS-miR-92a* and *UAS-miR-92b* transgenic flies were generated by Rainbow Transgenics.

The FRT-bearing transposon lines *e04431*, *e04047*, *e00089*, *d03337*, *e01786*, and *e03251* (Harvard Exelixis Collection) were used to generate FLP-FRT-based deletions, as described. Deletions were confirmed by PCR and sequencing.

In order to generate *Del #1*, *miR-92* ^*-/-*^ lines, which have one loxP site at *miR-92a* locus and two additional loxP sites at *miR-92b* locus, were used. Deletions were made as described in [16].

### Generation of *miR-92a* ^*–/–*^, *miR-92b* ^*–/–*^, and *miR-92* ^*–/–*^ Flies

Ends-out gene targeting was performed as described. Primers used to clone the 5’ and 3’ homology arms of miR-92a and miR-92b into pGX-attp are listed in S1 Table. The gene knockouts in mutant flies were confirmed by PCR, sequencing, and northern blot analysis.

### Clonal Analysis

MARCM clones were generated as described before by crossing *hs-flp70*, *Tub-Gal4*, *UAS-mCD8*:*GFP; FRT82B*, *Tub-Gal80* flies with *FRT82B* flies to generate control clones and with *FRT82B*, *miR-92* ^*4*^ flies to generate mutant clones. Clones were induced at 24 ± 6 hr ALH by heat shock in 37°C water bath for an hour and analyzed 72 hr after clone induction. The size of the clones was determined by counting the GFP^+^ cells in single clones. Type II clones were distinguished by presence of several small GFP^+^ Dpn^+^ cells (Intermediate progenitor cells) in addition to single large GFP^+^ Dpn^+^ neuroblast.

### qRT-PCR

For qRT-PCR, cDNA was synthesized from total RNA with random primers and superscript-II reverse transcriptase (Invitrogen). SYBR Green PCR Master Mix (Applied Biosystems) was used for real-time thermal cycling. The reactions were performed with a StepOne Plus Real-Time PCR System (Applied Biosystems) and StepOne software v2.1. *rp49* primers were used for normalization, and relative expression levels were calculated with the comparative CT method. The oligonucleotides used for qRT-PCR are listed in S1 Table.

For quantification of mature miR-92a and miR-92b, miRNA Taqman assay was used (Applied Biosystems assay number 000285 for miR-92a and 000286 for miR-92b). U6 was used as an internal reference.

### 3’RACE

Total RNA was extracted from larval brains with miRNeasy (Qiagen). 3’RACE was performed as described [38]. Two specific PCR bands of different sizes were gel purified with the QIAquick gel extraction kit (Qiagen), cloned into pGEMT-easy (Promega), and sequenced with vector-specific primers.

### Immunoblot Analysis

For western blot, 25 larval brains were dissected and lysed in 200 μl of RIPA buffer. Larval lysate (20 μl) was mixed with 2X Laemmli buffer, boiled, and separated by 12% SDS-PAGE. The blot was probed with rabbit anti-jigr1 (1:5000), generated against the N-terminal 110 amino acids of jigr1 protein by SDIX. Mouse anti-tubulin (T6199, Sigma 1:5000) was used as a loading control. Horseradish peroxidase–conjugated secondary antibodies were from Jackson ImmunoResearch Laboratories.

### Constructs and Mutagenesis

To generate miR-92a and miR-92b expression vectors, *Drosophila* pri-miR-92a and pri-miR-92b were amplified from genomic DNA and cloned into pSuper-GFP vector. MiR-124 used as a control miRNA. A pri-miR-124 expression plasmid was generated by amplification of 500 bp of primary sequence from mouse genomic DNA and cloning into pSuper-GFP vector.

To generate *jigr1* expression constructs, *jigr1* coding region containing short 3’UTR and long 3’UTR are amplified from cDNA and cloned into pUASt vector.

To generate a *jigr1* 3’UTR sensor, *jigr1* short and long 3’UTR sequences were amplified from genomic DNA and cloned into psiCHECK-2 downstream of *Renilla luciferase*. The target sites were mutated by site-directed mutagenesis with QuickChange multi and the QuickChange II site-directed mutagenesis kit (Stratagene). For the *Jigr1*-5’UTR sensor, oligonucleotides containing wild type and mutated miR-92a and miR-92b binding sites were synthesized, annealed, and cloned at the 5’ of the Renilla luciferase gene using the NheI site in psiCHECK-2 vector. The sequences of the wild type and mutant oligonucleotides are listed in S2 Table.

### BrdU Incorporation Assay

Drosophila third instar larvae were fed on food containing 1 mg/mL BrdU for 3 hours. Larval brains were immediately dissected, fixed and stained with the addition of a 2N HCl treatment for 30 min prior to BrdU staining.

For BrdU chase experiments, first clones were induced in *hs-flp70*, *Tub-Gal4*, *UAS-mCD8*:*GFP; FRT82B*, *Tub-Gal80* /*FRT82+* and, *hs-flp70*, *Tub-Gal4*, *UAS-mCD8*:*GFP; FRT82B*, *Tub-Gal80*/ *FRT82B*, *miR-92* ^*4*^ larvae at 24 ± 6 hr ALH. 48 hr later the larvae were first transferred to food containing 1mg/mL BrdU for 4 hours then to normal food for 24hr. Larval brains were dissected, fixed and stained with the addition of a 2N HCl treatment for 30 min prior to BrdU staining.

### Northern Blot Analysis

Northern blot for miRNAs was performed as described [39]. The following LNA modified probes from Exiqon were used. miR-92a: 5’-ATAGGCCGGGACAAGTGCAATG-3’; miR-92b: 5’-GCAGGCCGGGACTAGTGCAATT-3’; U6: 5’-CACGAATTTGCGTGTCATCCTT-3’.

Total RNA was extracted with the miRNAeasy mini kit (Qiagen). RNA (10–20 μg) was loaded onto a 15% polyacrylamide gel (Sequagel, National Diagnostics), transferred to Amersham Hybond-N^+^ (GE Healthcare), and crosslinked to the membrane with UV crosslinker. Hybridization was done overnight (16 hours) after 1 hour of pre-hybridization in DIG easy Hyb granules (Roche). For detection, DIG wash and block buffer set (Roche) were used according to the manufacturer’s protocol.

For northern blot analysis of *jigr1*, 10 μg of total RNA was loaded onto an agarose gel (1% agarose, 1x MOPS, and 5% formaldehyde) and transferred to a nylon membrane overnight by capillary transfer. Hybridization and detection were done as described above. To generate probes for *jigr1* and *rp49*, we amplified a ~500-bp region from genomic DNA by PCR. A T7 promoter sequence was added 5’ of the reverse primer. The probes were synthesized by in vitro transcription with T7 RNA polymerase (Roche). The oligonucleotides used to synthesize the *jigr1* and *rp49* probes are listed in S1 Table.

### RNA In Situ Hybridization

Third instar larval brains were dissected and fixed in 4% paraformaldehyde overnight at 4°C. The brains were transferred into 30% sucrose and incubated at 4°C overnight. Then, they were frozen and stored at -80°C until further use. Then 15 um sections were made. 5’ and 3’ DIG-labeled LNA-modified miR-92a and miR-92b probes (Exiqon) were used as recommended by the manufacturer. Anti-DIG-POD primary antibody (Roche; 1:500) was used and detected with the TSA Plus Cyanine 3 System (Perkin Elmer).

### Immunostaining and Confocal Imaging

Larval brains were dissected and fixed in 4% paraformaldehyde in phosphate-buffered saline for 20 minutes. The primary antibodies used were rabbit anti-Dpn (1:500) from Y.-N. Jan (University of California, San Francisco), mouse anti–Dlg 4F3 (1:20) from the Developmental Studies Hybridoma Bank (DSHB), rat anti-Miranda (1:500) from Chris Doe (University of Oregon), mouse anti-Prospero MR1A (1:20) from DSHB, rat anti-BrdU (1:500) from Abcam, goat anti-HRP-Cy3 from Jackson ImmunoResearch Laboratories, and mouse anti-nc82 (1:5) from DSHB. Secondary antibodies were from Life Technologies. Confocal images were taken with a Nikon D-Eclipse C1 and processed with Image J and Adobe Photoshop.

### Luciferase Assay

HEK293T cells were transfected with wild type or mutant miR-92 sensor plasmids or empty psiCHECK-2, pSuper-GFP-pri-miR-92a, or pSuper-GFP-pri-miR-92b expression vectors (described above) or pSuper-GFp-miR-124 using Fugene6 (Promega). 48 hours after transfection, cells were lysed in 1x passive lysis buffer (Promega). The Dual-Glo Luciferase Assay System from Promega was used according to the manufacturer’s protocol. Renilla luciferase activity was normalized to firefly luciferase activity. All the experiments were done in triplicate in three independent experiments.

## Supporting Information

S1 Fig Nucleotide sequence alignment and expression of miR-92a and miR-92b. (A) Sequence alignment of miR-92a and miR-92b mature sequences from different species (miRBase). (B) Quantification of mature miR-92a and miR-92b at different developmental stages of *Drosophila* by miRNA Taqman assay.
(TIF)

S2 Fig Expression of miR-92a and miR-92b in third instar larval brains. (A) Coexpression of miR-92a (red) with the neuroepithelial marker Discs large (green) in third instar larval brains. Scale bar: 20 μm. (B) miR-92a expression (red) in wild type, *miR-92b* ^*-/-*^ and *miR-92a* ^*-/-*^ third instar larval brains. Enlarged view of dashed box shows expression of miR-92a in Dpn^+^ neuroblasts. Scale bar: 20 μm and 5 μm. (C) miR-92b expression (red) in wild type, *miR-92a* ^*-/-*^ and *miR-92b* ^*-/-*^ third instar larval brains. Enlarged view of dashed box shows expression of miR-92b in Dpn^+^ neuroblasts. Scale bar: 20 μm and 5 μm.
(TIF)

S3 Fig Presence of *jigr1* extended 3’UTR in L3 larval brains. (A) Summary of all the deletions in the *jigr1* locus generated in this study. (B) Schematic representation of *jigr1* locus indicating the location of the primers used for 3’RACE. (C) DNA gel electrophoresis of RACE nested PCR products. (D) RNA-seq data obtained from modEncode project showing the expression of *jigr1* extended 3’ UTR in L3 CNS neuroblasts.
(TIF)

S4 Fig Life span analysis. Survival curve of male flies of the wild type (n = 100), *miR-92a* ^*–/–*^(n = 100), *miR-92b* ^*–/–*^(n = 100) and *miR-92* ^*–/–*^(n = 100) mutants.
(TIF)

S5 Fig Analysis of *miR-92* ^*-/-*^ mutant neuroblasts in third instar larval brains. (A) Single confocal sections of wild type and *miR-92* ^*–/–*^ third instar larval brain. Immunostaining of neuroblasts for Dpn (red) and cell cortex for Dlg (green). Scale bar: 20 μm. (B) Number of brain neuroblasts at 96 hr ALH. 1: *w* ^*1118*^ (n = 12); 2: *miR-92a* ^*-/-*^; 3: *miR-92b* ^*-/-*^; 4: *miR-92* ^*4/4*^ (n = 11); 5: *miR-92* ^*1/4*^ (n = 16); 6: *Insc-GAL4/UAS-miR-92a; miR-92* ^*4/4*^ (n = 10); 7: *Insc-GAL4/UAS-miR-92b; miR-92* ^*4/4*^ (n = 12); 8: *Insc-GAL4/UAS-miR-92a; miR-92* ^*1/4*^ (n = 8); 9: *Insc-GAL4/UAS-miR-92b; miR-92* ^*1/4*^ (n = 8). Statistical significance was determined by one-way ANOVA. (C) Quantification of apoptotic neuroblasts in the brains of wild type (n = 20) and *miR-92* ^*–/–*^(n = 18) third instar larvae. Statistical significance was determined by one-way ANOVA. (D) Neuroblast number in the brains of *miR-92* ^*1/4*^ and *Insc-GAL4*/*UAS-p35* (n = 16); *miR-92* ^*1/4*^ (n = 10) flies. Student’s t test was used for statistical analysis. Values are mean ± s.e.m. in all graphs. *: p < 0.05, **: p < 0.005, ***: p < 0.001, ****: p < 0.0001. (E) Immunostaining of wild type and *miR-92* ^*–/–*^ mutant larval brains for Prospero (green) and Dpn (red) at 96 hr ALH. Single confocal section is shown. Scale bar: 10 μm.
(TIF)

S6 Fig Asymmetric cell division is not disrupted in *miR-92* ^*-/-*^ neuroblasts. (A) Single confocal sections of wild type and *miR-92* ^*–/–*^ third instar larval brain neuroblast, stained with Miranda (green) and Numb (red). (B) Single confocal sections of wild type and *miR-92* ^*–/–*^ third instar larval brain neuroblasts, immunostained with Miranda (green) and aPKC (red). (C) Wild type and *miR-92* ^*-/-*^ neuroblast cells stained with Miranda (green) and phospho-histone H3 (red). Neuroblasts are encircled with dashed line. (D) Neuroblast clones of wild type control and *miR-92* ^*4*^ mutants. Clones are marked with CD8::GFP (green). Yellow arrows mark the neuroblast cell. Yellow dashed lines indicate the position of clones. Single focal planes are shown.
(TIF)

S7 Fig Prospero mRNA is not a direct target of miR-92a and miR-92b. (A) Staining of third instar larval brains of *Pros-GAL4/+*, *Pros-GAL4/UAS-miR-92a* and *Pros-GAL4/UAS-miR-92b* with Prospero (blue) and Jigr1 (red). Single confocal sections are shown. (B) Sequence alignment of predicted *miR-92a* and *miR-92b* binding sites with the 3’UTR of *prospero* mRNA. The seed sequences of miR-92a and miR-92b are shown in blue. (C) Dual luciferase assay of HEK 293T cell lysates cotransfected with miR-92a, miR-92b or empty vectors together with psicheck2 construct containing the short or long 3’UTR of *prospero* mRNA. Bar graph shows normalized mean luciferase activity of cells transfected with miR-92 expression plasmid to that of cells transfected with empty plasmid from two independent experiments. Statistical significance was determined by one-way ANOVA. Values are mean ± s.e.m. in all graphs. *: p < 0.05, **: p < 0.005, ***: p < 0.001, ****: p < 0.0001.
(TIF)

S8 Fig Life span analysis. Survival curve of *w* ^*1118*^ (red), *miR-92* ^*-/-*^ (green), *Def(3R)BSC321/miR-92* ^*4*^ (orange), *Def(3R)BSC321/miR-92* ^*1*^ (yellow), *Elav-GAL4/UAS-jigr1* ^*RNAi*^ *;miR-92* ^*1/4*^ (blue), *Del #1/miR-92* ^*1*^ (brown) flies.
(TIF)

S1 Table List of oligonucleotides used in this study. (DOCX)

S2 Table List of oligonucleotides used in site directed mutagenesis. (DOCX)
