# Supplementary figures and images for: Downregulation of the Host Gene jigr1 by miR-92 Is Essential for Neuroblast Self-Renewal in Drosophila
Source: PLoS Genet. 2015 May 22;11(5):e1005264. doi: 10.1371/journal.pgen.1005264 (PMC4441384; doi:10.1371/journal.pgen.1005264)

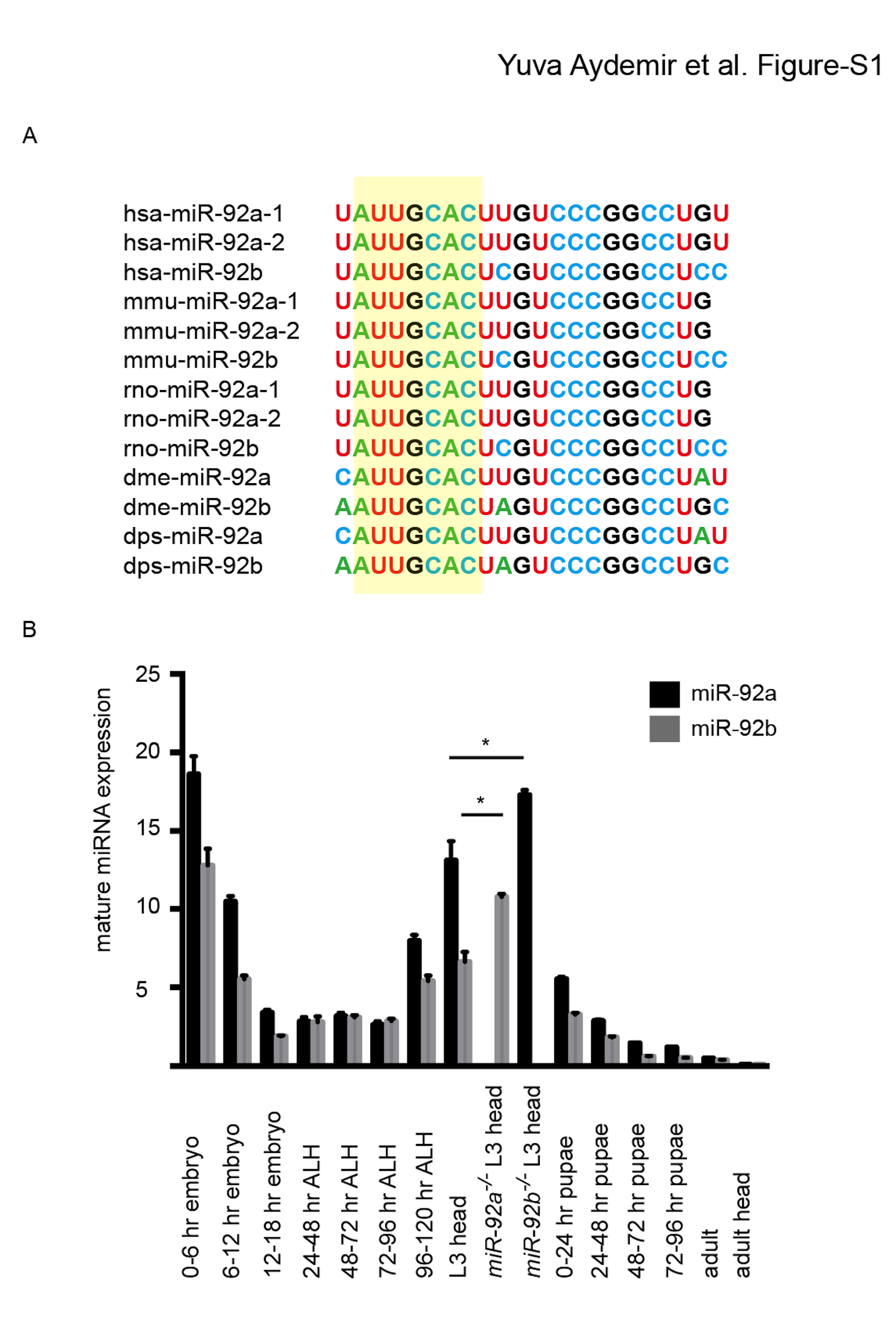

Supplement: S1 Fig — (A) Sequence alignment of miR-92a and miR-92b mature sequences from different species (miRBase). (B) Quantification of mature miR-92a and miR-92b at different developmental stages of Drosophila by miRNA Taqman assay. (TIF) [file pgen.1005264.s001.tif]

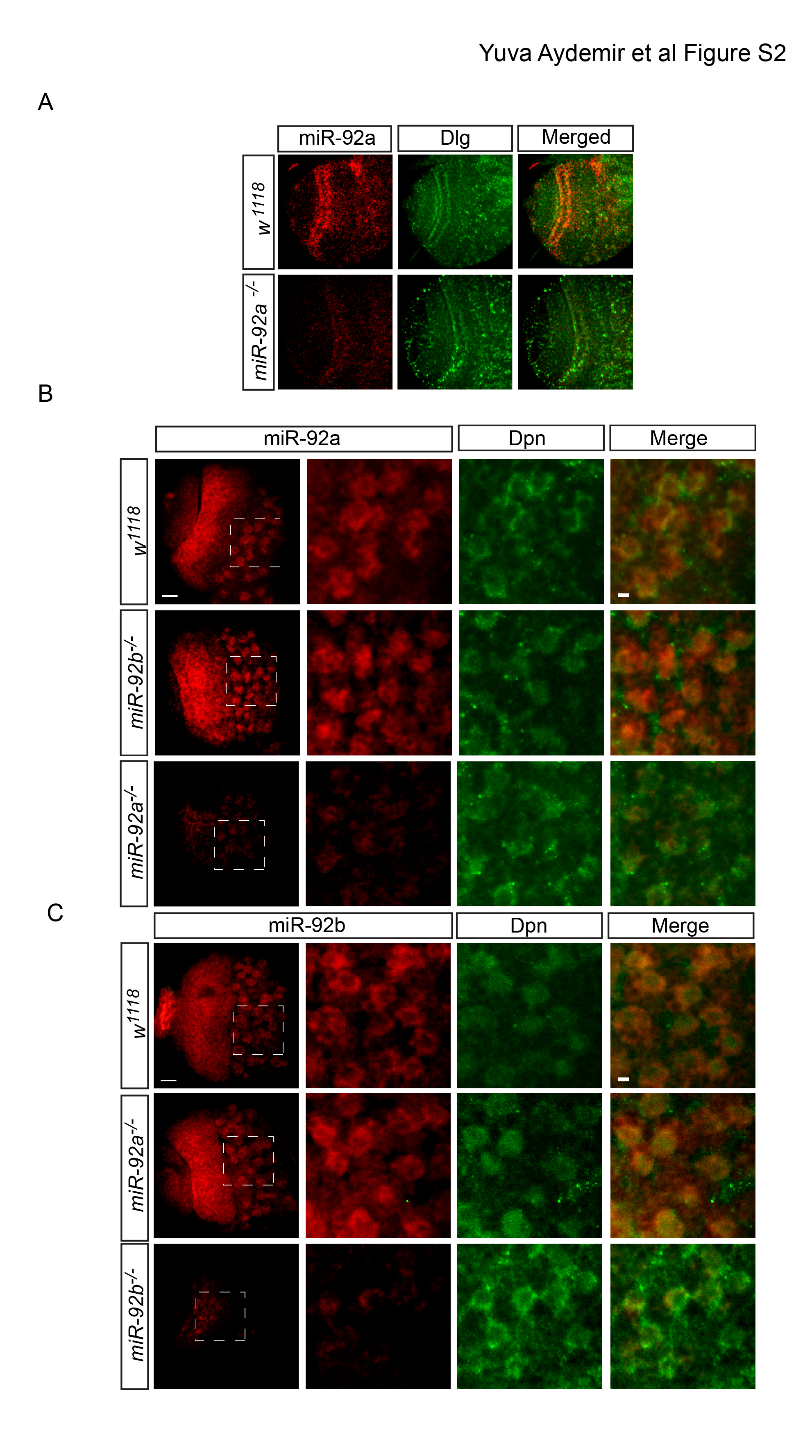

Supplement: S2 Fig — (A) Coexpression of miR-92a (red) with the neuroepithelial marker Discs large (green) in third instar larval brains. Scale bar: 20 μm. (B) miR-92a expression (red) in wild type, miR-92b -/- and miR-92a -/- third instar larval brains. Enlarged view of dashed box shows expression of miR-92a in Dpn+ neuroblasts. Scale bar: 20 μm and 5 μm. (C) miR-92b expression (red) in wild type, miR-92a -/- and miR-92b -/- third instar larval brains. Enlarged view of dashed box shows expression of miR-92b in Dpn+ neuroblasts. Scale bar: 20 μm and 5 μm. (TIF) [file pgen.1005264.s002.tif]

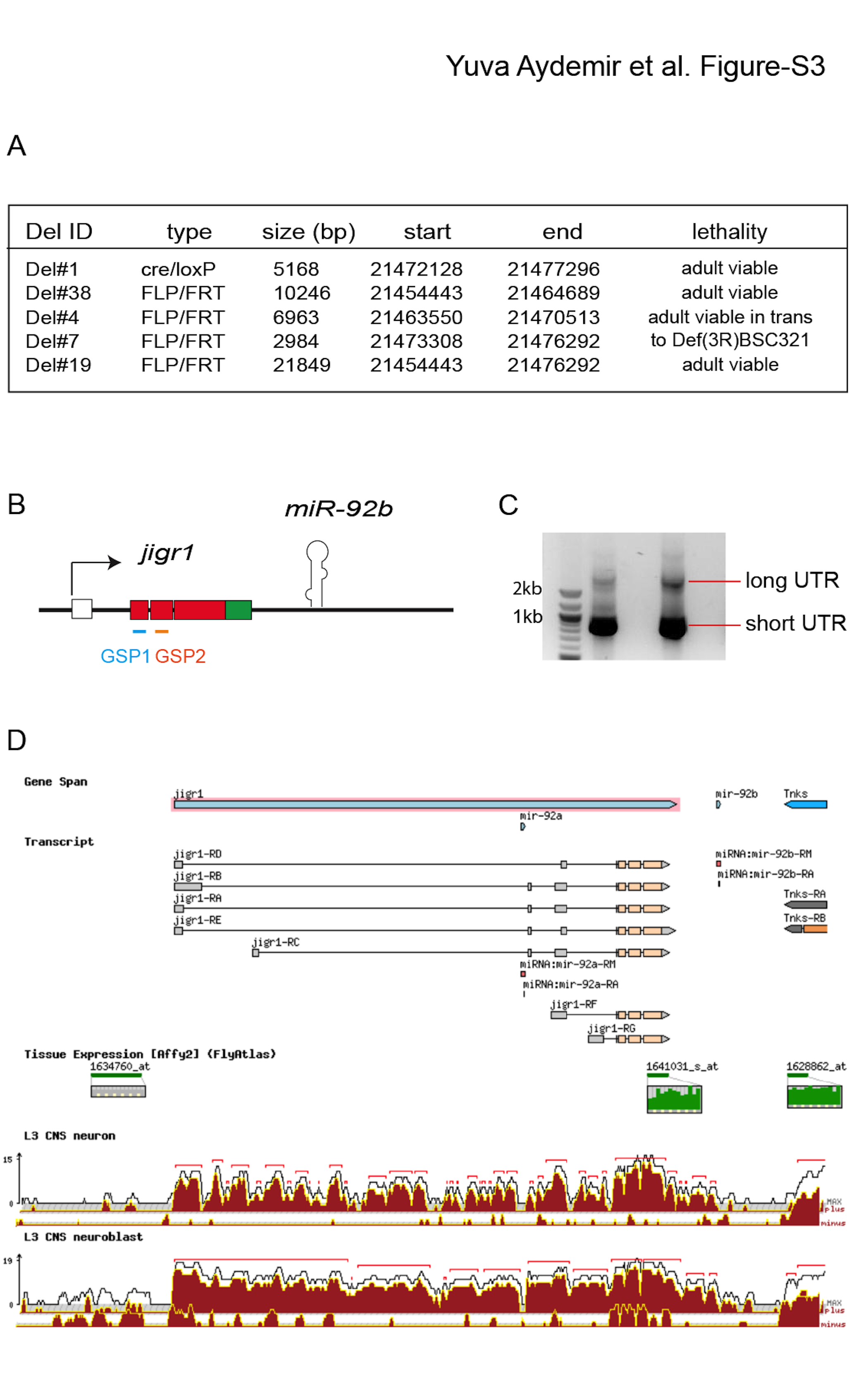

Supplement: S3 Fig — (A) Summary of all the deletions in the jigr1 locus generated in this study. (B) Schematic representation of jigr1 locus indicating the location of the primers used for 3’RACE. (C) DNA gel electrophoresis of RACE nested PCR products. (D) RNA-seq data obtained from modEncode project showing the expression of jigr1 extended 3’ UTR in L3 CNS neuroblasts. (TIF) [file pgen.1005264.s003.tif]

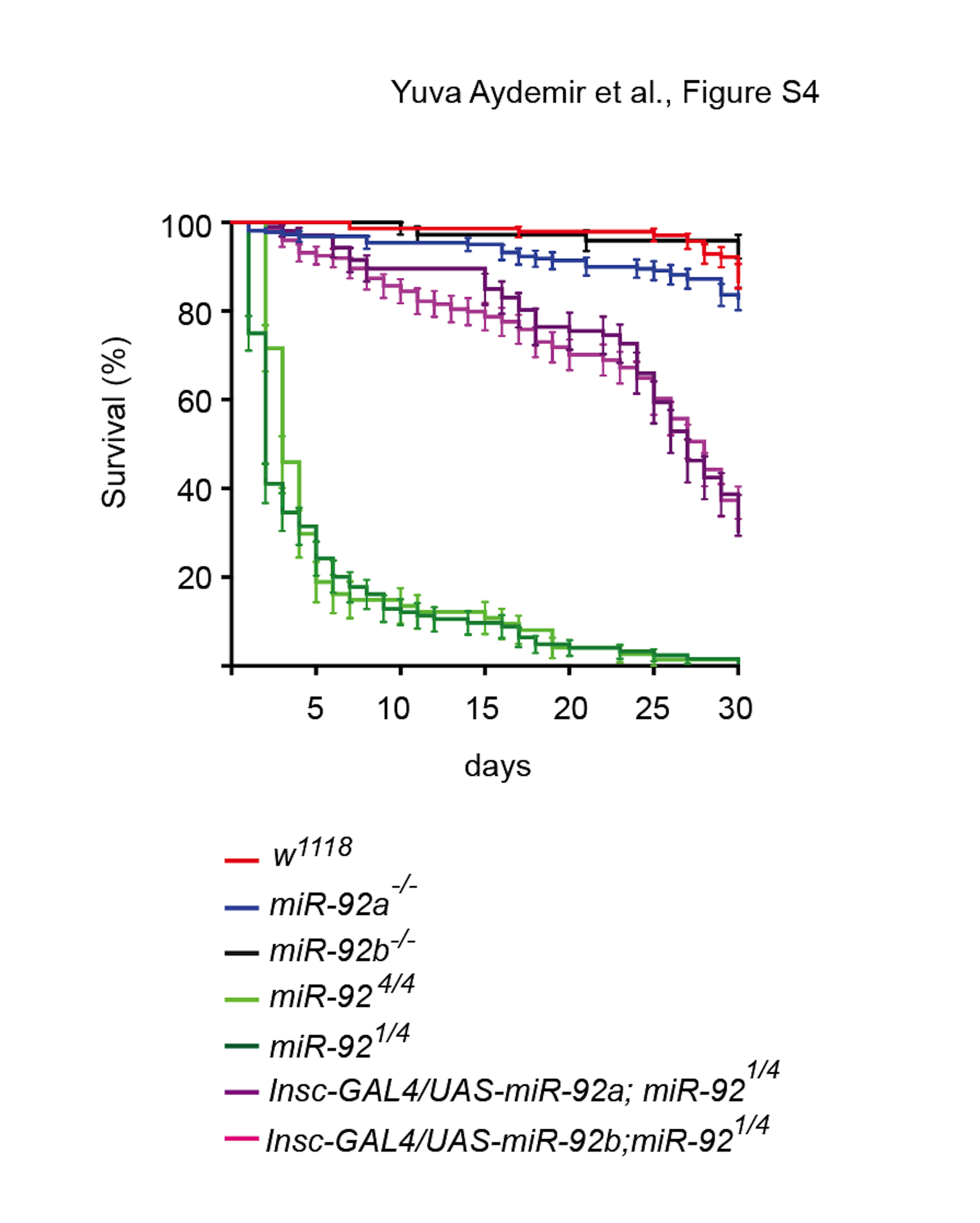

Supplement: S4 Fig — Survival curve of male flies of the wild type (n = 100), miR-92a –/–(n = 100), miR-92b –/–(n = 100) and miR-92 –/–(n = 100) mutants. (TIF) [file pgen.1005264.s004.tif]

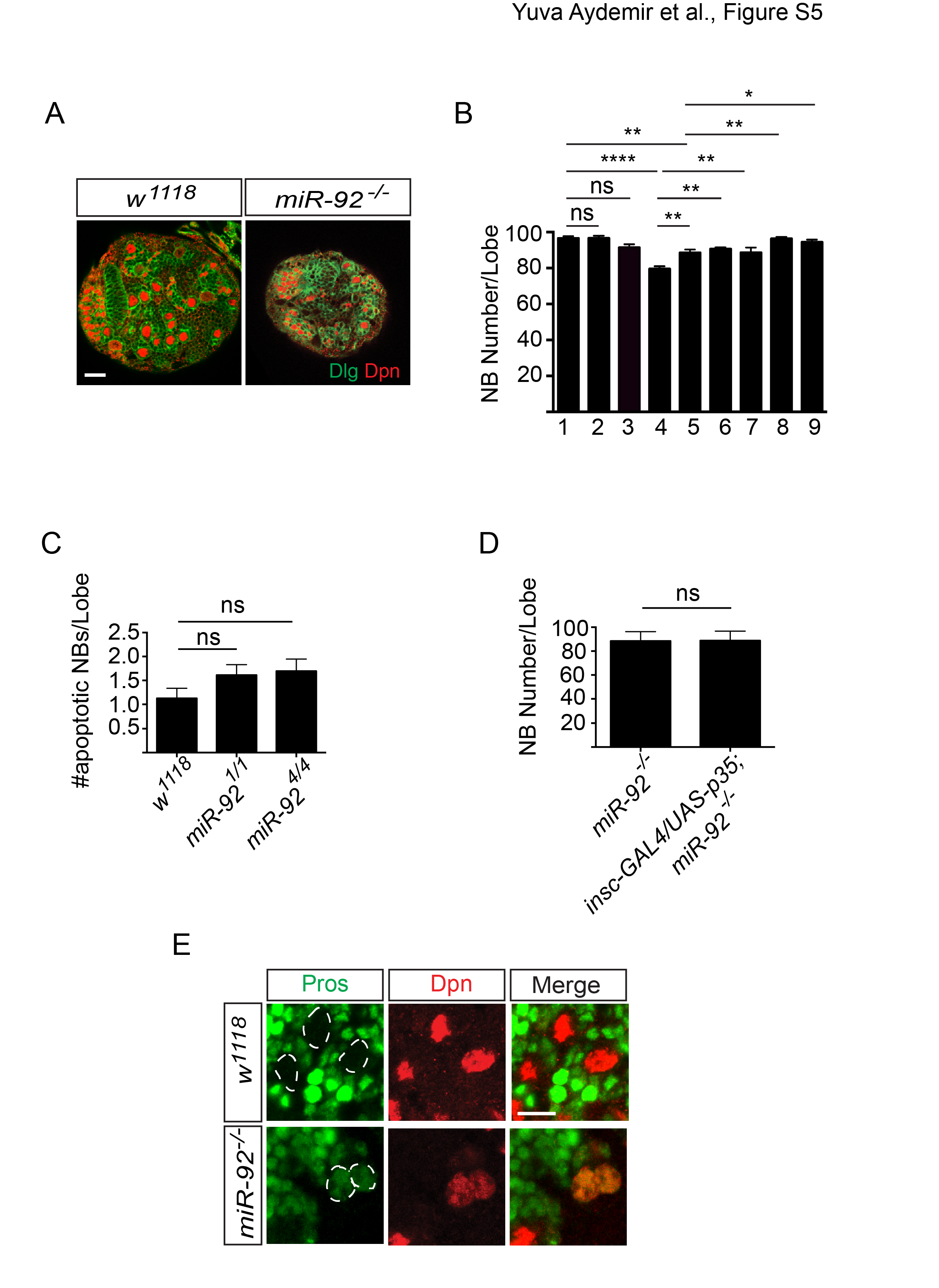

Supplement: S5 Fig — (A) Single confocal sections of wild type and miR-92 –/– third instar larval brain. Immunostaining of neuroblasts for Dpn (red) and cell cortex for Dlg (green). Scale bar: 20 μm. (B) Number of brain neuroblasts at 96 hr ALH. 1: w 1118 (n = 12); 2: miR-92a -/-; 3: miR-92b -/-; 4: miR-92 4/4 (n = 11); 5: miR-92 1/4 (n = 16); 6: Insc-GAL4/UAS-miR-92a; miR-92 4/4 (n = 10); 7: Insc-GAL4/UAS-miR-92b; miR-92 4/4 (n = 12); 8: Insc-GAL4/UAS-miR-92a; miR-92 1/4 (n = 8); 9: Insc-GAL4/UAS-miR-92b; miR-92 1/4 (n = 8). Statistical significance was determined by one-way ANOVA. (C) Quantification of apoptotic neuroblasts in the brains of wild type (n = 20) and miR-92 –/–(n = 18) third instar larvae. Statistical significance was determined by one-way ANOVA. (D) Neuroblast number in the brains of miR-92 1/4 and Insc-GAL4/UAS-p35 (n = 16); miR-92 1/4 (n = 10) flies. Student’s t test was used for statistical analysis. Values are mean ± s.e.m. in all graphs. *: p < 0.05, **: p < 0.005, ***: p < 0.001, ****: p < 0.0001. (E) Immunostaining of wild type and miR-92 –/– mutant larval brains for Prospero (green) and Dpn (red) at 96 hr ALH. Single confocal section is shown. Scale bar: 10 μm. (TIF) [file pgen.1005264.s005.tif]

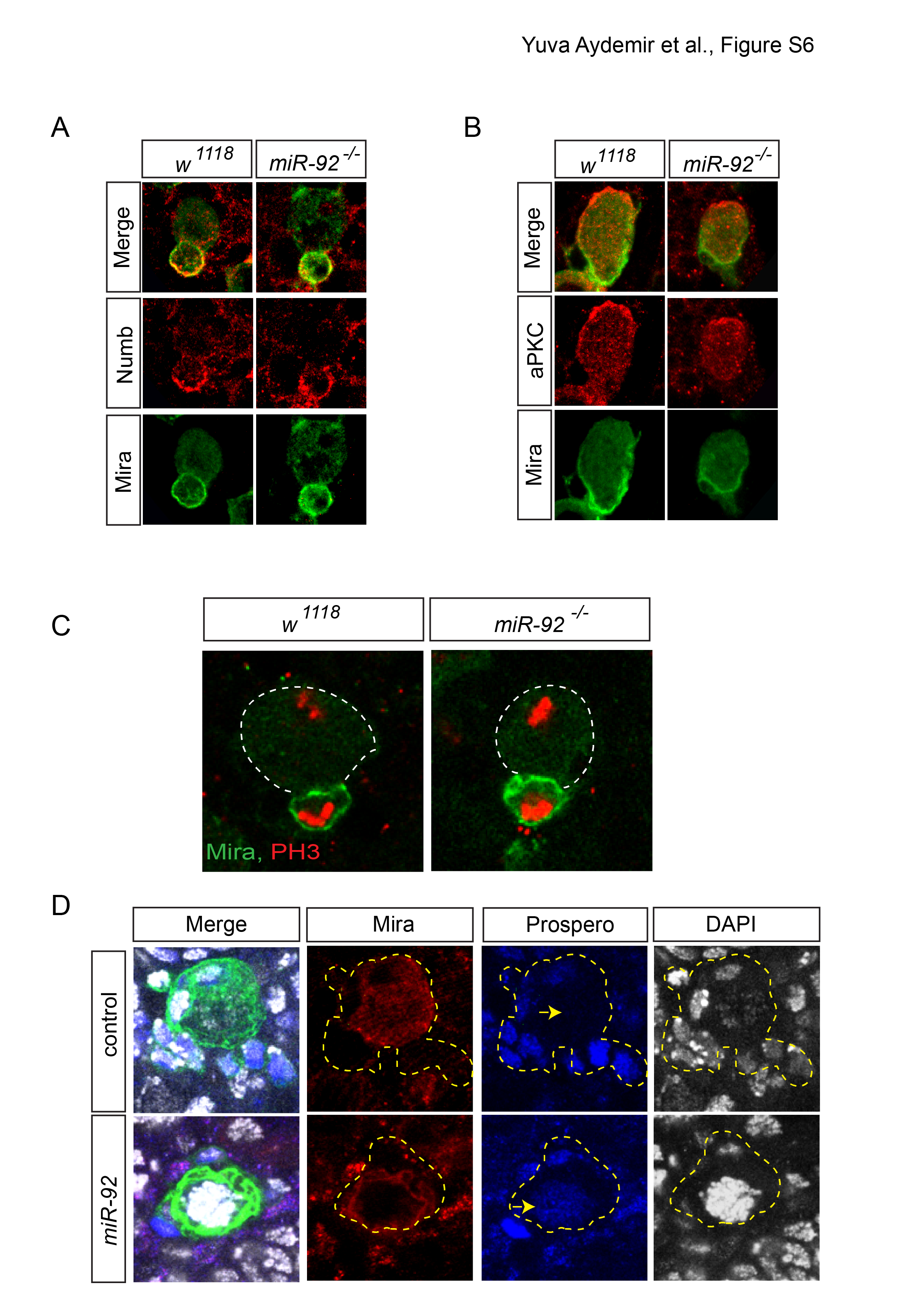

Supplement: S6 Fig — (A) Single confocal sections of wild type and miR-92 –/– third instar larval brain neuroblast, stained with Miranda (green) and Numb (red). (B) Single confocal sections of wild type and miR-92 –/– third instar larval brain neuroblasts, immunostained with Miranda (green) and aPKC (red). (C) Wild type and miR-92 -/- neuroblast cells stained with Miranda (green) and phospho-histone H3 (red). Neuroblasts are encircled with dashed line. (D) Neuroblast clones of wild type control and miR-92 4 mutants. Clones are marked with CD8::GFP (green). Yellow arrows mark the neuroblast cell. Yellow dashed lines indicate the position of clones. Single focal planes are shown. (TIF) [file pgen.1005264.s006.tif]

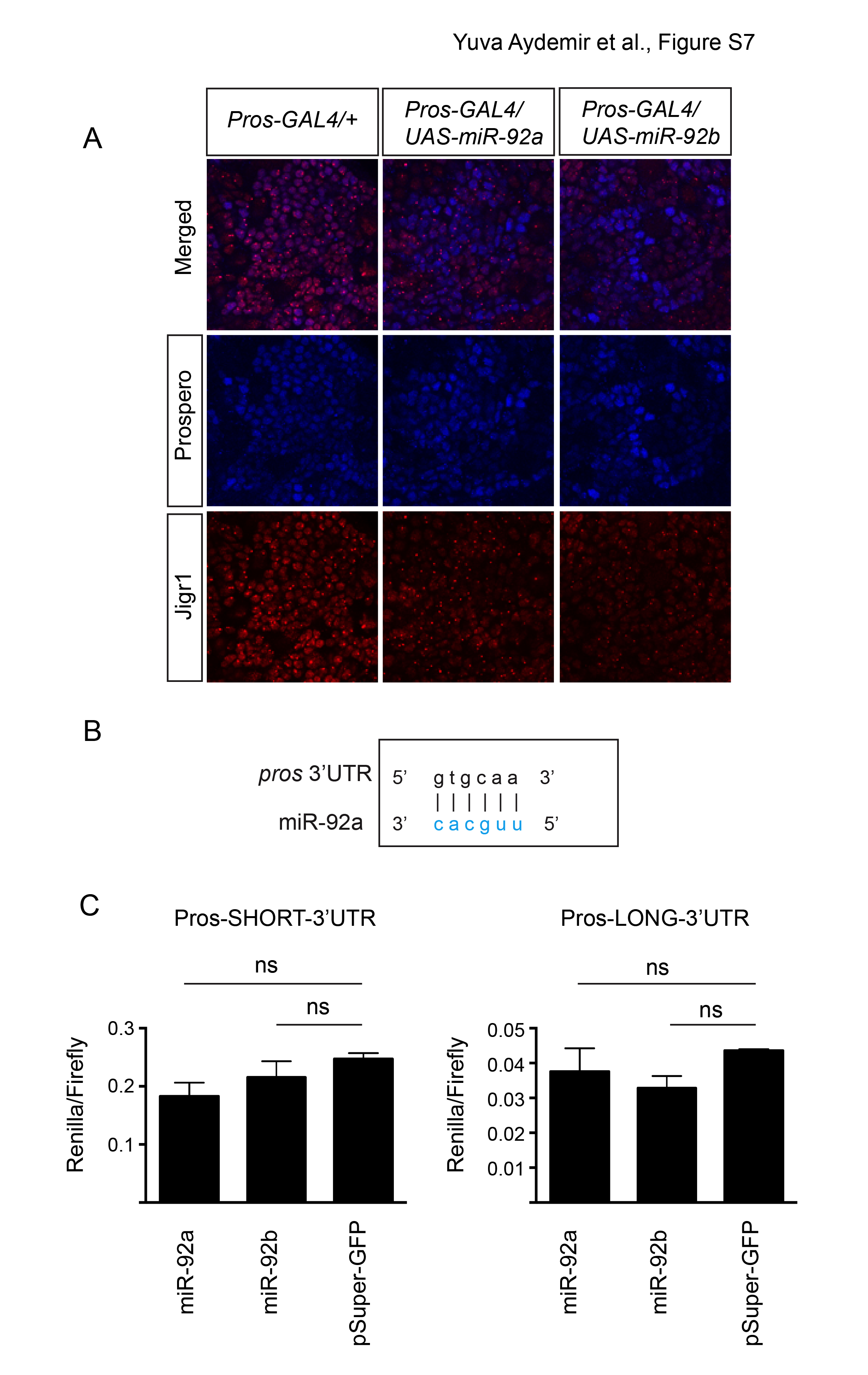

Supplement: S7 Fig — (A) Staining of third instar larval brains of Pros-GAL4/+, Pros-GAL4/UAS-miR-92a and Pros-GAL4/UAS-miR-92b with Prospero (blue) and Jigr1 (red). Single confocal sections are shown. (B) Sequence alignment of predicted miR-92a and miR-92b binding sites with the 3’UTR of prospero mRNA. The seed sequences of miR-92a and miR-92b are shown in blue. (C) Dual luciferase assay of HEK 293T cell lysates cotransfected with miR-92a, miR-92b or empty vectors together with psicheck2 construct containing the short or long 3’UTR of prospero mRNA. Bar graph shows normalized mean luciferase activity of cells transfected with miR-92 expression plasmid to that of cells transfected with empty plasmid from two independent experiments. Statistical significance was determined by one-way ANOVA. Values are mean ± s.e.m. in all graphs. *: p < 0.05, **: p < 0.005, ***: p < 0.001, ****: p < 0.0001. (TIF) [file pgen.1005264.s007.tif]

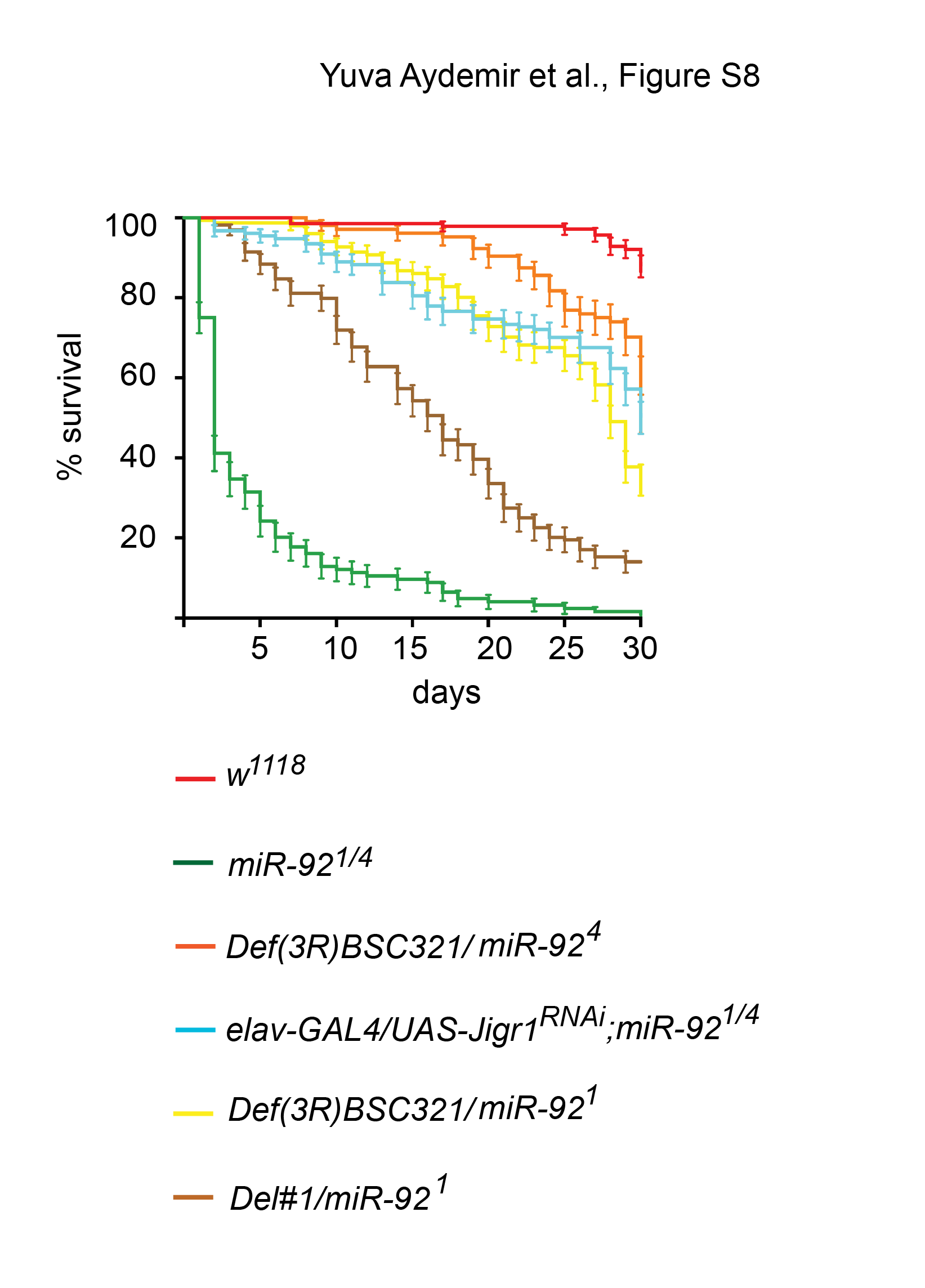

Supplement: S8 Fig — Survival curve of w 1118 (red), miR-92 -/- (green), Def(3R)BSC321/miR-92 4 (orange), Def(3R)BSC321/miR-92 1 (yellow), Elav-GAL4/UAS-jigr1 RNAi ;miR-92 1/4 (blue), Del #1/miR-92 1 (brown) flies. (TIF) [file pgen.1005264.s008.tif]
