# Supplementary material for: Downregulation of the Host Gene jigr1 by miR-92 Is Essential for Neuroblast Self-Renewal in Drosophila
Source: PLoS Genet. 2015 May 22;11(5):e1005264. doi: 10.1371/journal.pgen.1005264 (PMC4441384; doi:10.1371/journal.pgen.1005264)
Supplement: S1 Table — (DOCX) [file pgen.1005264.s009.docx]

**Table S1. Table S2 List of oligonucleotides used in site directed mutagenesis.**

|  | Sequence 5’–3’ | Notes |
| --- | --- | --- |
| 92a-up-F1 (SphI) | ACATGCATGCTTGCCGACGAAATCGAAATC | PCR primers for cloning miR-92a 5’ arm into pGX-attp |
| 92a-up-F2 (SphI) | ACATGCATGCTCGAATTCAAAACGCGTGTG |  |
| 92a-up-R1 (NheI) | CTAGCTAGCTCGGCTGAAAATCAGATCAC |  |
| 92a-up-R2 (NheI) | CTAGCTAGCTAGGCGAACAATAGGAGCATC |  |
| 92a-down-F1 (AscI) | TTGGCGCGCCTCTGTTGTTTCAGATCTCG | PCR primers for cloning miR-92a 3’ arm into pGX-attp |
| 92a-down-F2 (AscI) | TTGGCGCGCCGCAATATCAGAACATCTGTG |  |
| 92a-down-R4 (AvrII) | CCTAGGAACTGTCAAGTCGACCGAC |  |
| 92b-up-F1 (SphI) | ACATGCATGCACTTGTCAGCTGTTGGCTTG | PCR primers for cloning miR-92b 5’ arm into pGX-attp |
| 92b-up-F2 (SphI) | ACATGCATGCCCCTGCCAGCTTTTTCAATG |  |
| 92b-up-R1 (NheI) | CTAGCTAGCTGTACCACTGCACTCACATC |  |
| 92b-up-R2 (NheI) | CTAGCTAGCATGCAATCCGCTCCAAACTC |  |
| 92a-down-F1 (AscI) | TTGGCGCGCCACCACCATGGCCATAATGTG | PCR primers for cloning miR-92b 3’ arm into pGX-attp |
| 92a-down-F2(AscI) | TTGGCGCGCCTGCTGCTGTTCGTCAAAGTC |  |
| 92b-down-R1 (StuI) | GAAGGCCTACACTTCGATGTGACCAAGC |  |
| 92b-down-R2 (stuI) | GAAGGCCTATGTCAACGCCAACGATCTG |  |
| Vali-KO-92a-F | ACGAATTTGTCACGCTTTG | PCR primers for validation of targeting at miR-92a locus |
| Vali-KO-92a-R | TCGAGATCTGAAACAACAG |  |
| Vali-KO-R2 | ATTTCAAGGGTTTCCACTG |  |
| Vali-92b-KO-F | TCCATTTGACCAAACTGG | PCR primers for validation of targeting at miR-92b locus |
| Vali-92b-KO-R1 | CATTTTGGAGCTGATACG |  |
| Vali-KO-R2 | ATTTCAAGGGTTTCCACTG |  |
| 92a-KO-confirm-F | TACCTGCTCCCGATCCTATG | PCR primers for miR-92a deletion confirmation |
| 92a-KO-confirm-R | CACGAATTGAAAGCGATTGA |  |
| 92b-KO-confirm-F | ATTCCTGGCTCCTCTCTTCC | PCR primers for miR-92b deletion confirmation |
| 92b-KO-confirm-R | ATTACAGGGCCAGACATTCG |  |
| Pri-miR-92b–F1 | CACCATTGGGCAGTTTGGAGTTTG | PCR primers for cloning pri-miR-92b into pTW vector |
| Pri-miR-92b-R1 | GGCCATGGTGGTTAGCATTA |  |
| e04431-gp-F | GTGTTTCCATGTCCCATTCC | PCR primers for confirming deletion lines (Parks et al., 2004) |
| e04047-gp-R | CGGAAATGCGAGGATTAAGA |  |
| e00089-gp-R | GTGATTCTGGGTGGCCTTTA |  |
| RB3’minus-R | CCTCGATATACAGACCGATAAAAC |  |
| RB5’minus-F | TCCAAGCGGCGACTGAGATG |  |
| XP5’PLUS-F | AATGATTCGCAGTGGAAGGCT |  |
| RB3’PLUS-R | TGCATTTGCCTTTCGCCTTAT |  |
| XP5’PLUS-R | AGCCTTCCACTGCGAATCATT |  |
| RB3’PLUS-R | ATAAGGCGAAAGGCAAATGCA |  |
| d03337-del-confirm-F | CGTCAGACTCCACGACTTGA |  |
| Jigr1-ex6-probe-F | AATTCCCCTGAATAACTCTGACG | PCR primers for probe synthesis against exon6 of *jigr1* |
| Jigr1-ex6-probe-T7-R | TAATACGACTCACTATAGGGGATCTACCGGATCGACCTCA |  |
| Jigr1-longUTR-probe-F | GAACGGGTCGACTAATCCAA | PCR primers for probe synthesis against long-UTR of *jigr1* |
| Jigr1-longUTR-probe-T7-R | TAATACGACTCACTATAGGGTGATTCTGGGTGGCCTTTA |  |
| Rp49-probe-F | CCCAAGGGTATCGACAACAG | PCR primers for probe synthesis against rp49 |
| Rp49-probe-T7-R | TAATACGACTCACTATAGGGTTCCGACCACGTTACAGAA |  |
| JIGR1-int2-3-probe2-F | ATTAAAATGCCCGACCGTGG | PCR primers for probe synthesis against intron 2-3 of *jigr1* |
| JIGR1-int2-3-probe2-T7-R | TAATACGACTCACTATAGGGCTCACACAGACACACAACCG |  |
| jigr1-qPCR-F | TGTTAGGGATTCCCCTGAATAAC | qRT-PCR primers |
| jigr1-qPCR-R | TCCGCAAAGTGATTGTAGCC |  |
| rp49-qPCR-F | AGATCGTGAAGAA GCGCACCAAG | qRT-PCR primers |
| rp49-qPCR-R | CACCAGGAACTTCTTGAAATCCGG |  |
| HindIII-miR-92a-F | GCGCGCAAGCTTACCTGCCTACCTGTTG ATGG | PCR primers for cloning pri-miR-92a into pSuper-GFP |
| XhoI-miR-92a-R | CCGCTCGAGGAACACGAATTGAAAGCGATTG |  |
| HindIII-miR-92b-F | GCGCGCAAGCTTATTGGGCAGTTTGGAGTTTG | PCR primers for cloning pri-miR-92b into pSuper-GFP |
| XhoI-miR-92b-R | CCGCTCGAGTGGCCATGGTGGTTAGCAT |  |
| XhoI-jigr1-UTR-F | CCGCTCGAGGAGGTCGATCCGGTAGATCA | PCR primers for cloning short and long UTR of *jigr1* into psicheck2 vector |
| NotI-jigr1-longUTR-R | ATAAGAATGCGGCCGCGCTCCAGTGCGTCTCTTTTT |  |
| NotI-jigr1-shortUTR-R | ATAAGAATGCGGCCGCTTGGATTAGTCGACCCGTC |  |
| jigr1-longUTR-mut-site1 | CAGTTGAACTGTAGGCCATGAATAATGGCCGGGATAGCTGACCATCTAATAAACTAT | Mutagenesis primers |
| jigr1-longUTR-mut-site2 | CCTCCGGGCCATTTCTATGGCGGTTGCCCCCTTGTT |  |
| Q_T_ | CCAGTGAGCAGAGTGACGAGGACTCGAGCTCAAGCTTTTTTTTTTTTTTTTT | [37]  3’ RACE |
| Q_0_ | CCAGTGAGCAGAGTGACG |  |
| Q_I_ | GAGGACTCGAGCTCAAGC |  |
| Jigr1-GSP1 | GGGAACAGATAGCCCACAAA | 3’RACE |
| Jigr1-GSP2 | ACCTACGAGGTGGACGATTG |  |
| pros-UTR-PmeI-F  pros-UTR-NotI-R | GCAAGTTTAAACTGGAATAAGTGGAGGAGTTG  ATAAGAATGCGGCCGCGTGTGGCCGAAACTGCAATT | Luciferase assay |
