# Supplementary material for: Downregulation of the Host Gene jigr1 by miR-92 Is Essential for Neuroblast Self-Renewal in Drosophila
Source: PLoS Genet. 2015 May 22;11(5):e1005264. doi: 10.1371/journal.pgen.1005264 (PMC4441384; doi:10.1371/journal.pgen.1005264)
Supplement: S2 Table — (DOCX) [file pgen.1005264.s010.docx]

**Table S2. List of oligonucleotides used in site directed mutagenesis.**

|  | Sequence 5’–3’ |
| --- | --- |
| *Jigr1*-wt-5’ UTR sensor-F | CTAGCGCTGCCACCTGGTTGGCTGTGTGCTAGAGATGTGCAATTGAATGGTCTGTGCAATGAATAAAAAGTGTCCCAATGGTGCAATGCAGTTTAAAATGATTAAAAACGAAAAGTTAATA |
| *Jigr1*-wt-5’ UTR  sensor-R | CTAGTATTAACTTTTCGTTTTTAATCATTTTAAACTGCATTGCACCATTGGGACACTTTTTATTCATTGCACAGACCATTCAATTGCACATCTCTAGCACACAGCCAACCAGGTGGCAGCG |
| *Jigr1*-mut-5’ UTR  sensor-F | CTAGCGCTGCCACCTGGTTGGCTGTGTGCTAGAGATTGTAACGTGAATGGTCTTGTAACGGAATAAAAAGTGTCCCAATGTGTAACGGCAGTTTAAAATGATTAAAAACGAAAAGTTA |
| *Jigr1*-mut-5’UTR sensor-R | CTAGTATTAACTTTTCGTTTTTAATCATTTTAAACTGCCGTTACACATTGGGACACTTTTTATTCCGTTACAAGACCATTCACGTTACAATCTCTAGCACACAGCCAACCAGGTGGCAGCG |

Note: miR-92a and miR-92b binding sites are underlined. Mutations introduced are shown in

red and NheI restriction site is shown in blue.
